# Systematic analysis of the binding behaviour of UHRF1 towards different methyl- and carboxylcytosine modification patterns at CpG dyads

**DOI:** 10.1371/journal.pone.0229144

**Published:** 2020-02-21

**Authors:** Markus Schneider, Carina Trummer, Andreas Stengl, Peng Zhang, Aleksandra Szwagierczak, M. Cristina Cardoso, Heinrich Leonhardt, Christina Bauer, Iris Antes

**Affiliations:** 1 Center for Integrated Protein Science Munich at the TUM School of Life Sciences, Technische Universität München, Freising, Germany; 2 Center for Integrated Protein Science Munich at the Department of Biology II, Ludwig Maximilians University Munich, Planegg-Martinsried, Germany; 3 Cell Biology and Epigenetics at the Department of Biology, Technische Universität Darmstadt, Darmstadt, Germany; Universität Stuttgart, GERMANY

## Abstract

The multi-domain protein UHRF1 is essential for DNA methylation maintenance and binds DNA via a base-flipping mechanism with a preference for hemi-methylated CpG sites. We investigated its binding to hemi- and symmetrically modified DNA containing either 5-methylcytosine (mC), 5-hydroxymethylcytosine (hmC), 5-formylcytosine (fC), or 5-carboxylcytosine (caC). Our experimental results indicate that UHRF1 binds symmetrically carboxylated and hybrid methylated/carboxylated CpG dyads in addition to its previously reported substrates. Complementary molecular dynamics simulations provide a possible mechanistic explanation of how the protein could differentiate between modification patterns. First, we observe different local binding modes in the nucleotide binding pocket as well as the protein’s NKR finger. Second, both DNA modification sites are coupled through key residues within the NKR finger, suggesting a communication pathway affecting protein-DNA binding for carboxylcytosine modifications. Our results suggest a possible additional function of the hemi-methylation reader UHRF1 through binding of carboxylated CpG sites. This opens the possibility of new biological roles of UHRF1 beyond DNA methylation maintenance and of oxidised methylcytosine derivates in epigenetic regulation.

## Introduction

**Fig 1:**
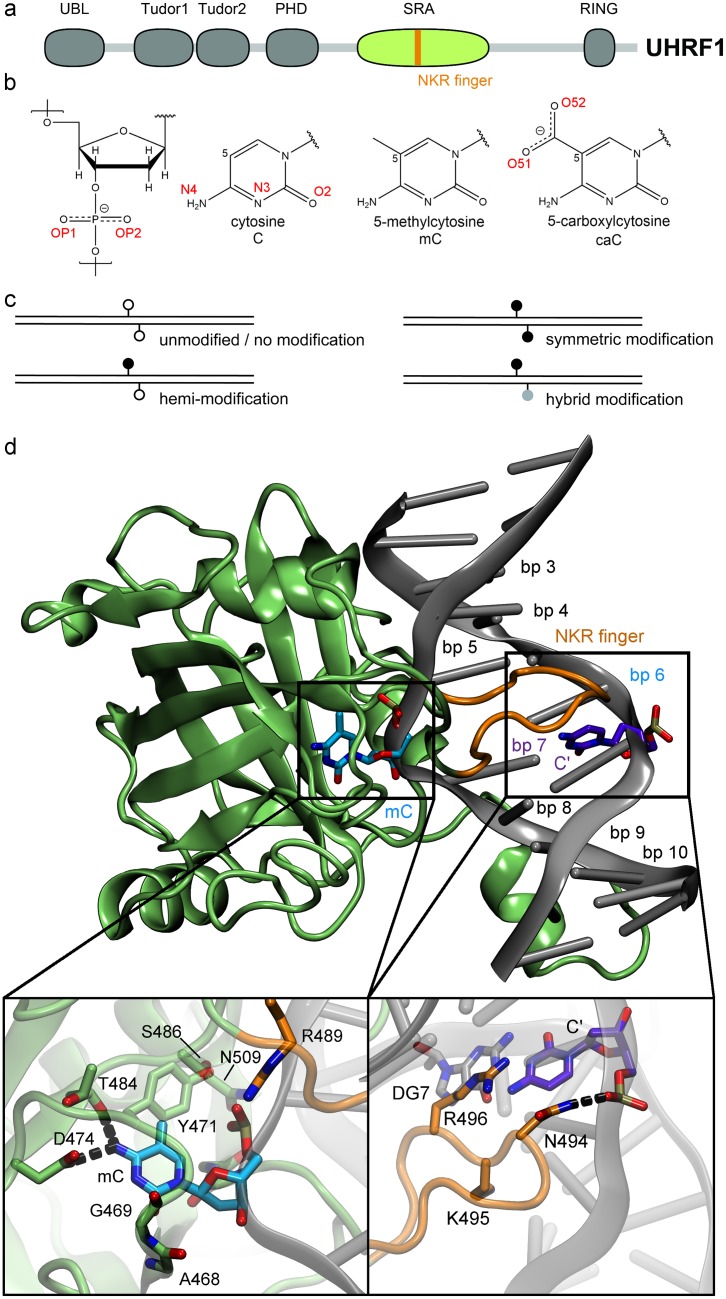
Structure of the UHRF1—DNA complex. (a) Schematic structure of UHRF1. The Tudor-like domains and the PHD-type zinc finger recognize the histone marks H3K9me2/3 and H3R2me0, respectively, while the SRA domain (in green, also referred to as YDG domain) is important for DNA binding. (b) Chemical structure and atom names of the modified DNA bases methylcytosine (mC) and carboxylcytosine (caC). (c) Schematic illustration of possible cytosine modification configurations on CpG dyads. (d) Representative molecular dynamics structure of the SRA domain of UHRF1 bound to hemi-methylated DNA. Insets show a magnification of the nucleotide binding pocket and NKR finger regions. DNA base pairs (bp) are numbered based on the strand binding the flipped-out base.

UHRF1 (also referred to as Np95) is an essential protein for DNA methylation maintenance in mammals. It consists of 5 domains: A ubiquitin-like domain, a Tandem-Tudor domain, a PHD domain, a DNA-binding SRA domain, and a RING domain with E3 ubiquitin ligase activity (Fig 1a) [1–3]. UHRF1 was originally reported to preferentially bind to hemi-methylated DNA, i.e. DNA harbouring 5-methylcytosine (mC) only on one strand. Upon binding of the methylated strand, UHRF1 recruits DNA methyltransferase 1 (DNMT1) for additional methylation of the second strand, yielding a symmetrically methylated CpG site [1–3]. This recruitment depends on specific histone ubiquitination, set by the RING domain of UHRF1 and recognized by a ubiquitin interaction motif of DNMT1 [4–6].

Besides mC, three other cytosine (C) modifications exist in mammalian cells, i.e. 5-hydroxymethylcytosine (hmC), 5-formylcytosine (fC), and 5-carboxylcytosine (caC) [7–9]. These variants are generated by the family of TET proteins through step-wise oxidation of mC and are discussed to be either intermediates in active DNA demethylation or independent epigenetic marks [10]. Their overall abundance in vivo is normally magnitudes lower than that of methylated sites [11], but the ratio increases under certain conditions. Higher hmC concentrations were observed in neuronal cells [12], while a study investigating breast and glioma tumour tissues found that a substantial portion of the samples exhibited increased caC levels [13]. Efforts to map mC, hmC, fC, and caC modifications in the genome showed that they accumulate at functionally distinct regions of transcription regulation [14–16]. One common conclusion of these studies was that methylation/demethylation of CpG sites is a highly dynamic and genome-wide process. In this light, low concentrations of some DNA modifications could represent a transient state in a high turnover process, while the accumulation at functionally diverse sites suggests that some variants might have a biological role beyond being demethylation intermediates. It has been demonstrated that several proteins recognize some oxidised variants with similar or even greater affinity than mC. The UHRF family member UHRF2, which features a highly similar domain architecture to UHRF1 [17, 18], is a reader with increased affinity for hmC in neuronal progenitor cells [19]. Other examples include SUVH5, which binds both mC and hmC with similar strength [20], while POL II, WT1 and TET3 specifically recognize caC [21–23]. It is currently unclear how frequent certain CpG modification patterns occur in vivo. DNA replication during S-phase will generally result in hemi-modified CpG sites. In case of mC, the subsequent restoration of the DNA modification to symmetry is well studied and described [24]. Nevertheless, the degree of persistent hemi-methylation varies between cell types and genomic elements [25]. For hmC, fC, and caC, no maintenance pathways have been described so far. In vitro, TET proteins predominantly generate symmetric fC sites [26], whereas genomic mapping approaches suggest the existence of hmC and fC/caC in hemi-modified form [15, 27]. The occurrence of hybrid modifications with mC on one and an oxidised cytosine derivative on the other strand is also likely (Fig 1c).

Structural analysis revealed that the SRA domain of UHRF1 flips the methylated cytosine out of the DNA strand and envelopes it within its binding pocket. In addition, the protein binds to the DNA by inserting its thumb region into the minor groove and its NKR finger region into the major groove [2, 28, 29]. In a previous work, our groups showed by a combination of in vitro experiments and molecular dynamics (MD) simulations that UHRF1 binds hemi-modified hmC with similar affinity as hemi-mC [30]. Although subsequent studies revealed that UHRF1 binds hmC with lower affinity than mC, it still binds hmC with 1.3 to 3-fold higher affinity than unmodified C [19, 31, 32]. These results are in line with an unbiased mass spectrometry screen for epigenetic readers in embryonic stem cells, which demonstrated UHRF1 binds to all modified cytosines, but in particular to mC and hmC [19]. Experiments with UHRF1 and symmetrically modified mC sites, i.e. CpG sites in which both DNA strands feature methylcytosine, consistently show reduced binding affinity [1, 2, 28, 29]. This selectivity is commonly explained by a hydrogen bond between N494 at the tip of the NKR finger and the C’ cytosine, i.e. the base that potentially carries the symmetric modification (Fig 1d) [29]. Throughout the manuscript we use a terminal apostrophe to mark bases on the distal DNA strand (e.g. C’). Bianchi et al. observed in a computational study that the presence of mC on both strands sterically impairs binding of the NKR finger of UHRF1 to the major groove [33]. In contrast to mC and hmC, the structural effects of fC and caC variants on UHRF1-DNA binding are still not well elucidated. Investigations of several SRA domains by Rajakumara et al. suggest a reduced affinity of UHRF1 towards hemi-hmC, -fC and–caC containing DNA [20]. Crystal structures of POL II and TDG, which exhibit specific activity towards caC, show that the caC carboxyl group participates in specific hydrogen bond networks, which are crucial for binding key recognition residues in the protein [21, 34].

It was recently shown that UHRF1 allosterically regulates its activity and binding properties through intramolecular conformational changes [35–38]. The formation of these extensive inter-domain interactions illustrates an inherent flexibility of UHRF1 and allows the protein to adapt to different substrates. As we already observed solid binding of UHRF1 to hemi-hmC, we sought to systematically analyse the binding behaviour of UHRF1 towards CpG sites containing C, mC, hmC, fC, and caC either in a hemi-, hybrid or symmetrically modified state. The highest binding affinities are observed for hemi-mC, symmetric caC, and the caC-mC’ hybrid. To understand the differences in recognition of these modifications, we performed molecular dynamics simulations of mC- and caC-modified DNA in complex with the SRA domain of UHRF1 (see Fig 1d).

## Materials & methods

### Electrophoretic mobility shift assays (EMSAs)

Expression constructs for GFP-mUHRF1 and mUHRF2-GFP have been described previously [18, 39]. In general, protein purification and EMSAs were performed as reported in Spruijt et al. [19]. Briefly, a 2-fold serial dilution of protein (300 nM to 4.69 nM) in binding buffer (including 100 ng/μl BSA final concentration) was incubated with a 1:1 mixture of two fluorescently labelled 42 bp oligonucleotides (Eurofins Genomics) at a stable concentration of 250 nM each. After 30 min of incubation on ice, reactions were run over a 6% native PAGE in 0.5x TBE buffer (45 mM Tris-borate, 1 mM EDTA). ATTO647N-labelled DNA (“C^647^") served as internal control and reference whereas ATTO550-labelled DNA carried one of the following cytosine variants at the central CG site: canonical C, mC, hmC, fC, or caC (“xC^550^”). Fluorescent signal was detected with a Typhoon Trio+ scanner (GE Healthcare Life Sciences). Signal of bound and unbound fractions were quantified with ImageJ by plotting the mean grey values per lane and measuring the area under the selected peaks. Before quantitation, gel pictures were assigned random names to blind the experimenter during analysis. Box plots show $\frac{ATTO550\;bound\;fraction}{ATTO647\;bound\;fraction}\times \frac{ATTO647\;total\;signal}{ATTO\;550\;total\;signal}$ with the C^550^/C^647^ experiment as control. All raw gel image scans with annotations are provided as S1 Fig.

### Microscale Thermophoresis (MST)

For MST, the SRA domain of mouse UHRF1 (residues 419–628) was cloned into a hexahistidine-tagged construct and protein was expressed in Escherichia coli BL21(DE3)-Gold cells (Stratagene). The purified SRA domain was labelled with a NT-647 dye using the Monolith NT^™^ His-Tag Labelling Kit RED-tris-NTA (NanoTemper Technologies) according to the manufacturer’s instructions and 50 nM of the labelled protein was incubated for 20 min at room temperature with increasing concentrations of the corresponding DNA oligonucleotide (C-C’, mC-C’, caC-C’, caC-caC’, mC-caC’) in PBS-T (0.05% Tween-20). The solutions were then aspirated into NT.115 Standard Treated Capillaries (NanoTemper Technologies) and placed into the Monolith NT.115 instrument (NanoTemper Technologies). Experiments were conducted with 60% LED power and 80% MST power. Obtained fluorescence signals were normalized (F_norm_) and the change in F_norm_ was plotted as a function of the concentration of the titrated binding partner using the MO. Affinity Analysis software version 2.3 (NanoTemper Technologies). For fluorescence normalization (F_norm_ = F_Hot_/F_cold_), the manual analysis mode was selected and cursors were set as follows: F_cold_ = -1 to 0, F_hot_ = 9 to 10 (see S2 Fig). Data of four to five independent measurements were analysed and means were fitted to obtain the respective K_D_ values. More detailed information and additional experimental procedures can be found in S1 Text.

### Force field parameterization of modified cytosine bases

We generated parameters for the parmbsc1 force field [40] for both deoxy-5-methylcytosine (mC) and deoxy-5-carboxylcytosine (caC) using the mC structure and bonded parameters template from Lankas et al. [41], which was originally derived for parmbsc0 [42]. The atom type of the C3’ atom was changed from CT to CE to adjust the template to parmbsc1. Fixed point atom charges were derived for both mC and caC following the procedure in ref. [43] using the R.E.D Dev webserver [44–48]. Atom types were assigned and final parameter files prepared using the programs antechamber and prepgen of the AmberTools17 package [49]. The final parameter files are provided in S1 File.

### Molecular dynamics simulations

Molecular dynamics simulations were performed with the Amber16/AmberTools17 software suite [49] using the Amber14SB force field for protein and parmbsc1 for nucleic acid parameters [40, 50]. All systems were based on the crystal structure of a mouse UHRF1 SRA domain bound to DNA featuring a single mC (PDB-ID: 3FDE). The same structure had been used in our previous work analysing the binding of 5-hydroxymethylcytosine [30] and featured the best resolution (1.41 Å) of published UHRF1 structures at the time of this study. Cytosine modifications were modelled and topologies prepared using leap (AmberTools). Each system was solvated in a box of TIP3P water [51] with a minimum face distance of 15 Å and 150 mM NaCl. A direct space cutoff of 12 Å was used for nonbonded potentials and PME summation was applied for electrostatic interactions. Energy minimization was performed until convergence to 0.01 kcal * mol^-1^ * Å^-1^ using the XMIN minimizer. Then, the volume of the solvent box was modified such that the density increased in 0.02 kg * m^3^ steps and energy minimization was repeated for each step until a target density of 1.00 kg * m^3^ was reached. For all molecular dynamics simulations hereafter, a time step of 1 fs and SHAKE [52] for bonds connected to hydrogens were used. The system was gradually heated from 0 to 300 K over 1.7 ns, applying a variation of the step-wise heatup protocol established within our group [53]. Within these steps, restraints of 2.39 kcal * mol^-1^ * Å^-2^ were applied to all heavy atoms until 20 K and on protein/DNA backbone atoms until 200 K. For heatup, a Langevin thermostat was used with a collision frequency of 4 ps^-1^, and for the last 0.5 ns a Berendsen barostat was employed with a relaxation time of 2 ps. During the following simulations at 300 K, a slow coupling Berendsen thermostat with a coupling time of 10 ps was used in combination with a Berendsen barostat and a respective relaxation time of 5 ps. Backbone phosphates and oxygens of terminal DNA residues were harmonically restrained with a constant of 2.39 kcal * mol^-1^ * Å^-2^ while resetting target coordinates in 500 ps intervals. For all replicas, different initial velocities and random seeds for the Langevin thermostat were generated at the beginning of each step of the heatup protocol (i.e. for each temperature simulated). Each replicon was simulated for 200 ns, yielding a total simulation time of 1 μs per system (5 replicas). In two out of thirty simulations (caC-caC’_r2 and mC-caC’_r2), the DNA structure diverged notably from the others (RMSD > 4 Å; see S3 and S4 Figs). In the case of caC-caC’_r2, the distortion correlates with an interaction between the protein’s free C-terminal helix and the DNA strand, bending it out of position, which is clearly an artefact due to the use of the isolated SRA domain. Therefore, and as it is in general difficult to determine whether such diverging trajectories show a rare but physically relevant conformational change or a simulation artefact, we excluded these two replicas from our analysis. The remaining simulations showed stable RMSD curves after about 20 ns. To allow for proper equilibration and to minimize any bias towards the initial structure, we extracted only the last 100 ns of each trajectory and afterwards merged the trajectories of all five replicas into a single system-specific trajectory that was used for all computational analyses.

Trajectory post-processing was performed with CPPTRAJ [54] version 17.00 unless otherwise indicated. Salt bridges were calculated using the “nativecontacts” command and a cutoff of 5 Å, saving both native and non-native time series and selecting interactions with opposite formal charges involving Arg, Lys, Glu, Asp and nucleotide residues. Hydrogen bonds were extracted using the “hbond” command, a cutoff distance of 4 Å and an angle cutoff of 120°. CPPTRAJ outputs were merged and converted into networks using our analysis tools AIFGen and CONAN (manuscript in preparation). Root mean square deviation (RMSD) and root mean square fluctuation (RMSF) calculations were performed for non-hydrogen atoms using the CPPTRAJ “rmsd” and “atomicfluct” commands after aligning each simulation frame to the protein’s Cα atoms without the terminal regions (residues 432 to 586). For RMSD, the reference frame was the simulation’s initial structure, while for RMSF the protein was aligned to its simulation average. DNA major and minor groove widths were calculated using the method of El Hassan and Calladine [55] as implemented in the “nastruct” command in CPPTRAJ (version 18.01). Figures of protein and DNA structures were prepared using VMD 1.9.3 [56]. Plots and supporting calculations (e.g. gaussian kernel estimates) were generated with matplotlib 2.0.0 [57].

## Results

### Experimental investigation of the binding behaviour of UHRF1 towards different cytosine variants

**Fig 2:**
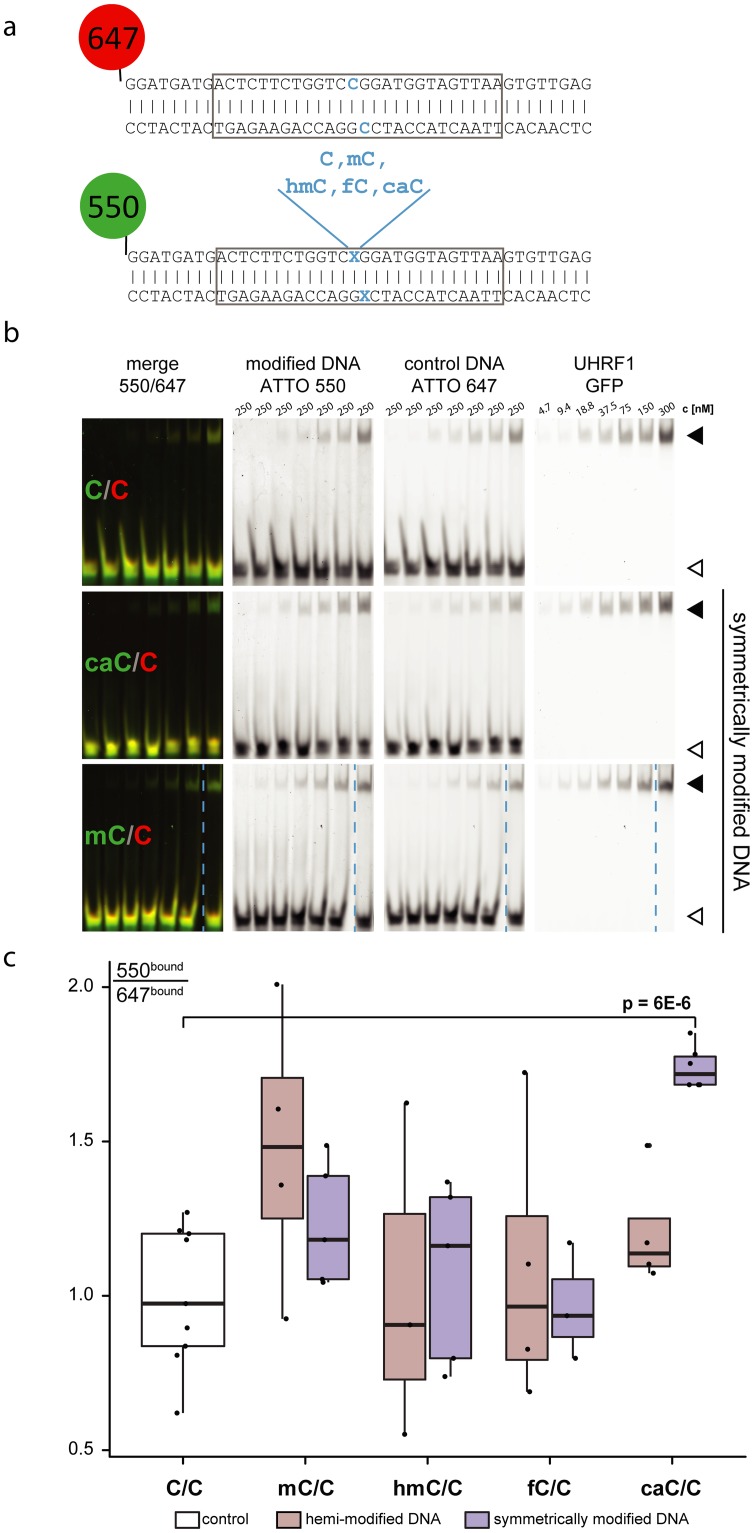
Binding of UHRF1 to differentially modified CpG sites. (a) DNA used in EMSA experiments. The 550-labelled DNA contains a central CG site harbouring different cytosine modifications: Unmodified C, mC, hmC, fC, or caC. The modification resides either on one strand (hemi-modification) or on both strands (symmetric modification). The 647-labelled oligonucleotide is always unmodified and serves as an internal control and reference. Grey boxes indicate sequences of the shorter DNA fragments used in Fig 3. (b) Representative images of EMSAs. Fluorescently labelled DNA oligonucleotides of 42 bp are incubated with GFP-UHRF1 at increasing protein concentrations. Black arrowheads indicate the DNA-protein complex (bound fraction); white arrowheads show free DNA. Dashed blue lines indicate empty gel lanes that have been removed for presentation purposes. (c) Quantitation of the bound fraction of symmetric and hemi-modified DNA incubated with wild type UHRF1, p value of two-tailed student’s t-test.

For systematic analysis of the binding specificities of UHRF1 towards the five known cytosine variants, we performed EMSA experiments with full-length UHRF1 in complex with 42 bp oligonucleotides harbouring C, mC, hmC, fC, or caC at a central CpG site (Fig 2a). To correct for general DNA binding affinity, two DNA fragments were used in direct competition in each EMSA experiment: A 647-labeled unmodified oligonucleotide and a 550-labeled oligonucleotide carrying the modification of interest in either hemi-modified (xC-C’) or symmetric (xC-xC’) state. 647-labeled unmodified DNA is used as internal control and reference for quantification. This allows direct comparison of UHRF1 binding affinity to all modifications without the need for pair-wise competition assays. Generally, EMSAs showed binding of UHRF1 to all studied DNA variants (example gel pictures are shown in Fig 2b). However, quantitation of the shifted fractions reveals a 1.5-fold preference for hemi-mC and a statistically significant 2-fold preference for symmetric caC (Fig 2c). All other modification variants, including hemi-caC, were bound with comparable strength to unmodified DNA. Similarly, we observed a 2-fold preference of UHRF2 for symmetric caC (S5 Fig).

Upon UHRF1 binding, the melting temperature of CpG-containing DNA is slightly reduced compared to its unbound state or a non-CpG-control, indicating a destabilization of the DNA duplex (S6a Fig). Complementary to our EMSA results, the SRA domain of UHRF1 substantially shifted the melting temperature of symmetrically carboxylated DNA to lower temperatures, whereas a weaker shift was observed for unmodified and hemi-methylated DNA (S6 Fig). To rule out that the thermal shift observed for symmetrically carboxylated DNA is due to different binding stoichiometries, we examined DNA-protein complex formation by size-exclusion chromatography. Binding of the SRA domain to the modified DNA oligonucleotides led to a comparable shift in retention time for all modifications tested (S7 Fig), indicating a uniform binding stoichiometry for UHRF1 independent of the DNA’s modification state.

**Fig 3:**
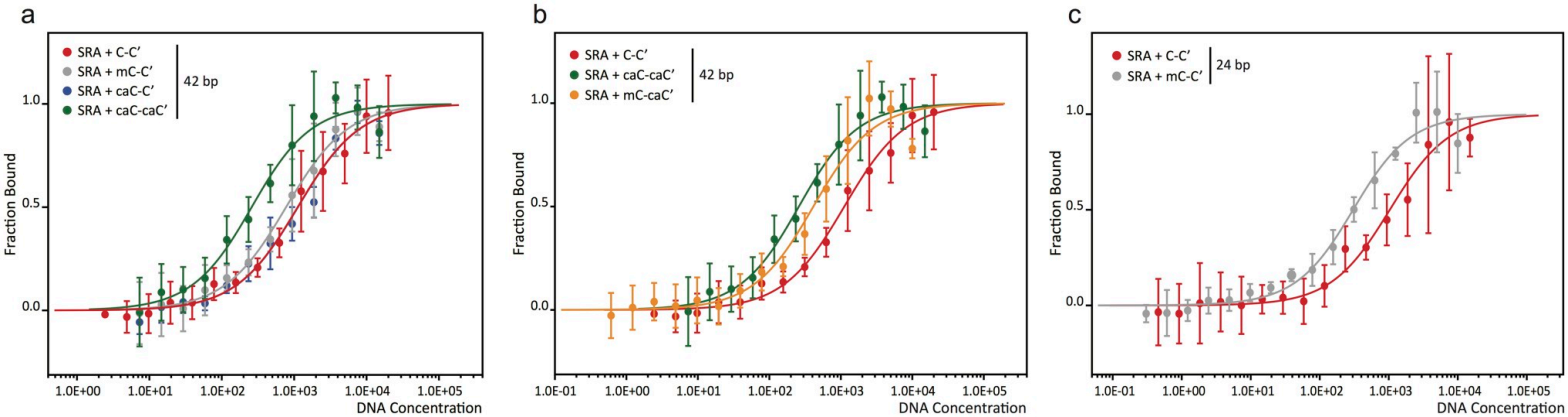
Microscale Thermophoresis experiments of UHRF1-SRA bound to DNA with modified CpG sites. (a,b) Dissociation constants of UHRF1 bound to a 42 bp DNA oligonucleotide: 1.10±0.15 μM for C-C’, 0.75±0.11 μM for mC-C’, 1.10±0.29 μM for caC-C’, 0.23±0.05 μM for caC-caC’, and 0.39±0.11 μM for mC-caC’. (c) Dissociation constants of UHRF1 bound to a 24 bp oligonucleotide; 1.01±0.20 μM for C-C’ and 0.28±0.06 μM for mC-C’. Curves show the fitted average values of 4–5 independent experiments.

To better characterize the binding of UHRF1 to hemi-mC, hemi-caC and symmetric caC, we determined the respective dissociation constants (K_D_) with Microscale Thermophoresis [58] (MST) experiments (Fig 3a). We observed slightly stronger binding of hemi-mC (K_D_ = 0.75±0.11 μM vs. 1.10±0.15 μM for unmodified DNA) and considerably enhanced binding of symmetric caC (K_D_ = 0.23±0.05 μM). In agreement with the EMSA results, hemi-carboxylated DNA (K_D_ = 1.10±0.29 μM) is bound with similar affinity as unmodified DNA. Taken together, we performed three independent experimental assays, i.e. EMSAs, melting temperature analysis and MST, which consistently confirm a binding preference of UHRF1 towards symmetric caC.

Additionally, as the enzymatic reactions involved in generation of mC and caC modifications suggest the potential existence of hybrid mC-caC’ sites, we determined the K_D_ of the SRA domain of UHRF1 and a mC-caC’ oligonucleotide and observed binding comparable to symmetric caC (K_D_ = 0.39±0.11 μM vs. 0.23±0.05 μM). In summary, UHRF1 exhibits a binding preference for caC modifications opposite of mC or caC, but not C.

Since the difference in K_D_ between unmodified and hemi-methylated DNA was smaller than expected from the literature [1, 32, 36, 59, 60], we repeated the MST experiments with shorter DNA oligonucleotides of 24 bp to reduce the number of unspecific binding sites (Fig 3c). With this new setup we observed a 3.6-fold preference of the SRA domain of UHRF1 towards hemi-methylated CpG sites (K_D_ = 0.28±0.06 μM for mC-C’ vs. 1.01±0.20 μM for C-C’). This ratio is in very good agreement with data by Greiner et al. [60] and Zhou et al. [32] (Table 1), who reported a 3.5 or 3.4-fold smaller K_D_ for hemi-methylated CpGs for a 12 bp oligonucleotide, respectively, compared to unmodified DNA. Generally, caution is advised when published K_D_ values of UHRF1 and differentially modified DNA are compared, since applied methods, DNA substrates and protein constructs used vary greatly among studies, resulting in a broad range of K_D_ values from 1.8 nM to 9.23 μM (Table 1). Nonetheless, previous studies and our results not only demonstrate the sensitivity of UHRF1 to different types of cytosine modification, but also the dependency of measured binding affinities on modification density, i.e. the number of DNA modifications compared to unmodified DNA stretches.

**Table 1:** Published K_D_ values for UHRF1 and DNA with differentially modified CpG sites.

Citation	Method	Affinity	DNA substrate	protein construct
Bostick, M. et al., 2007, 10.1126/science.1147939	EMSA	K_D_(mC-C’) = 1.8 nM	39mer, 13 modification sites	murine SRA
		K_D_(mC-mC’) = 12.1 nM		
Fang, J., 2016, 10.1038/ncomms11197	Fluorescence Polarization	K_D_(UHRF1) = 0.35 μM	12mer, 1 modification site	human UHRF1, different constructs with mC-C’
		K_D_(SRA) = 9.23 μM		
		K_D_(SRA+Spacer^a^) = 0.49 μM		
Greiner, V. J., 2015, 10.1021/acs.biochem.5b00419	FRET	K_D_(mC-C’) = 0.08 μM	12mer, 1 modification site	human SRA
		K_D_(mC-mC’) = 0.25 μM		
		K_D_(C-C‘) = 0.28 μM		
		K_D_(T-C‘) = 0.55 μM		
Qian, C., 2008, 10.1074/jbc.C800169200	Fluorescence Polarization	K_D_(mC-C’) = 0.2 μM	13mer, 1 modification site	human SRA
Zhou, T., 2014, 10.1016/j.molcel.2014.04.003	Fluorescence Polarization	K_D_(C-C’) = 8.61 μM	12mer, 1 modification site	human SRA
		K_D_(mC-C’) = 2.56 μM		
		K_D_(hmC-hmC’) = 7.97 μM		
Schneider, Trummer et al., 2019	MST	K_D_(C-C’) = 1.01 μM	24mer, 1 modification site	murine SRA
		K_D_(mC-C’) = 0.28 μM		
Schneider, Trummer et al., 2019	MST	K_D_(C-C’) = 1.10 μM	42mer, 1 modification site	murine SRA
		K_D_(mC-C’) = 0.75 μM		
		K_D_(caC-C’) = 1.10 μM		
		K_D_(caC-caC’) = 0.23 μM		
		K_D_(mC-caC’) = 0.39 μM		

^a^ Spacer: amino acid stretch C-terminal of SRA domain

### Molecular dynamics simulations of the UHRF1-SRA domain bound to CpG sites with mC and caC modifications

For methylated CpG sites, UHRF1 binds stronger to mC-C’ modified DNA than to the symmetric modification variant mC-mC’ (Table 1) [1, 60]. As discussed above, in our experiments the opposite was observed for caC modifications, as caC-caC’ DNA was preferred over caC-C’. To understand this behaviour, we performed MD simulations of UHRF1-DNA complexes with different nucleotide modifications, i.e. hemi-modified and symmetrically modified mC and caC as well as the hybrid modification variants mC-caC’ and caC-mC’. As simulation of the full binding process for all variants was not feasible due to the high complexity and computational cost of such simulations, we focused on studying the complex with the flipped-out modified base bound in the protein’s binding pocket, based on the experimental structure of mC-C’ bound to UHRF1 (PDB-ID: 3FDE). Various experimental data indicate that this is the most relevant state for recognition: Fluorescence kinetics experiments [61] showed that the stability of the DNA flipped state is correlated to the lifetime of the flipped state bound to protein. Regarding flipping propensity, previous simulation studies showed no substantial intrinsic difference between mC and caC [62] and furthermore, NMR experiments of Dickerson–Drew dodecamers showed that both mC and caC bases were slightly less likely to flip compared to unmodified cytosines [63]. Finally, in a study of another base-flipping protein, bacterial cytosine-5-methyltransferase, it was found that specific protein-base interactions were responsible for facilitating and stabilizing the flipped out state [64]. We chose to simulate the second potentially modified base on the distal strand in the flipped-in state, motivated by the following observations: First, stable flipping of the distal base has only been observed for proteins which can bind in a 2:1 protein-DNA ratio to the same CpG site, like UHRF2 or SUVH5, but not UHRF1 [2, 28, 29, 32, 65]. Second, the NKR finger can recognize modifications on the distal strand directly, as demonstrated by the crystal structure contacts of N494 [2, 29, 66] and third, it was observed that a single mutation of this residue abolishes the selectivity of UHRF1 between mC-C’ and mC-mC’ [29]. Finally, computational studies reported that the first stable intermediate in the flipping process requires a flip angle of at least 50° [62, 67]. It is difficult to imagine how direct interactions of the NKR finger could be sustained with the modified base in this position. For these reasons, we consider the complex conformation with a flipped-out pocket bound base and a flipped-in base on the distal DNA strand as the most relevant for explaining the selectivity of UHRF1.

Therefore, we did not aim at the simulation and analysis of the binding process itself and its related binding affinities, but rather at identifying similarities and differences in the binding modes of the different DNA modifications, i.e. which regions of the protein are likely to sense the chemical differences of these modification types and how this influences their interaction patterns. In contrast to mC, the caC modification contains an additional carboxyl group, which can form additional salt bridges and hydrogen bonds. Thus, we analysed whether this difference in interaction capacity could affect the polar interaction network and the local conformations of the binding pocket and NKR finger regions, which are in direct contact with the two modification sites.

**Fig 4:**
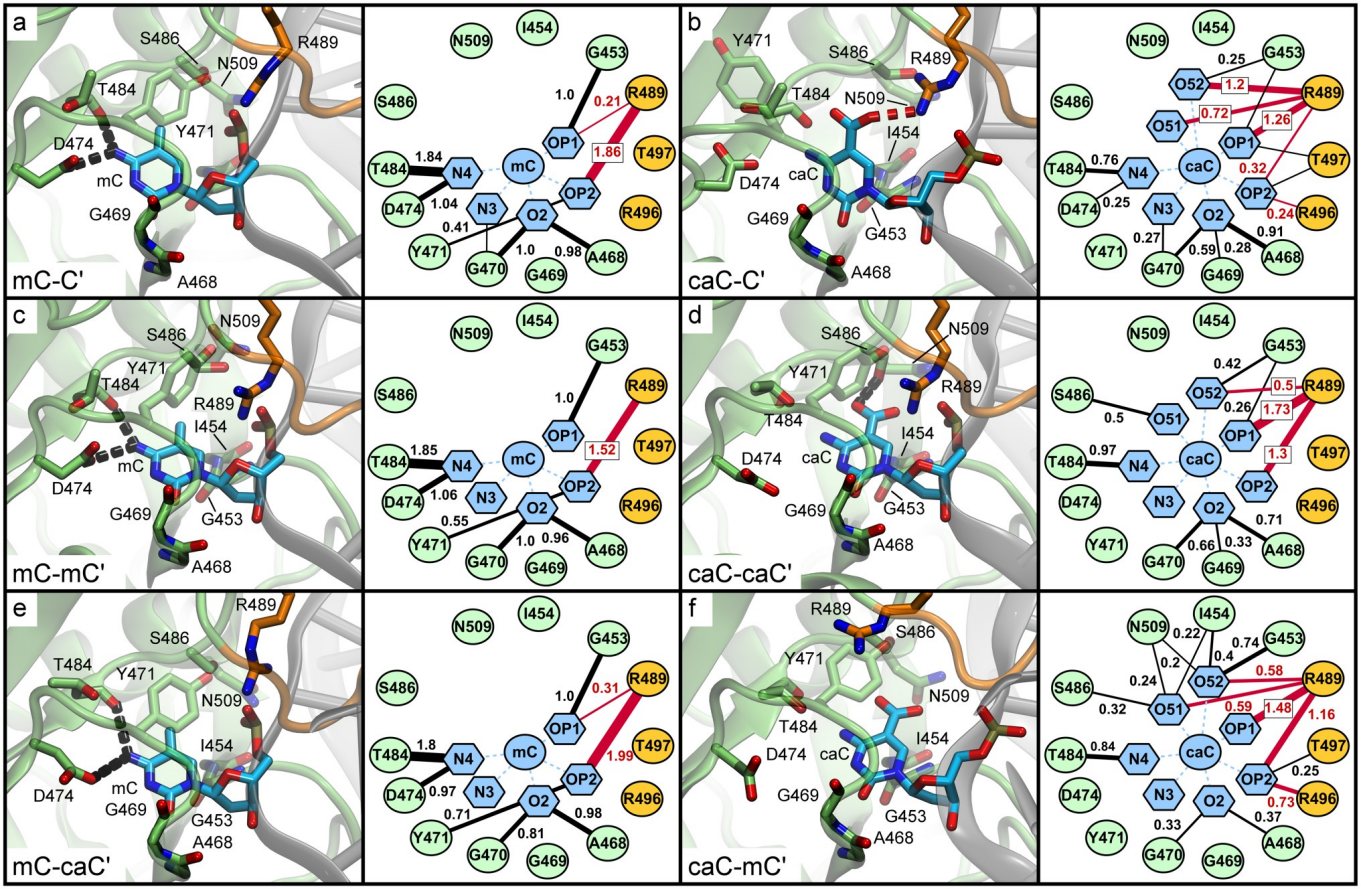
Interaction networks of the nucleotide binding pocket based on molecular dynamics simulations of UHRF1-SRA. Structures show representative conformations of the flipped-out modified DNA base within the binding pocket as observed during MD simulations. To the right of each structure a corresponding network of hydrogen bonds (black lines) and salt bridges (red lines) averaged over the course of the simulation is shown. Numbers next to edges show the average number of interactions per time frame. Edges representing interactions occurring in ≤ 15% of simulation time are omitted for clarity. For node pairs featuring both hydrogen bonds and salt bridges, only salt bridges are displayed.

Analysis of mC and caC recognition in the UHRF1-SRA nucleotide binding pocket. In Fig 4 we provide the interaction networks of the flipped base in the nucleotide binding pocket as derived from our MD simulations. Nodes represent residues of the protein and atoms of the modified DNA bases (see naming conventions in Fig 1b), while edges show the average number of hydrogen bonds (black lines) and salt bridges (red lines) between two nodes during the simulation. The canonical binding mode of mC-C’ (Fig 4a) is characterized by strong hydrogen bonds between the mC atom N4 to T484 and D474 (1.84 and 1.04 hydrogen bonds on average per analysed simulation frame, respectively) and between the pyrimidine oxygen O2 and G470 and A468 (1.0 and 0.98 hydrogen bonds on average). Thus, the base is effectively locked at these two positions with the N4 and O2 atoms acting as handles. In addition, the mC backbone atom OP1 (phosphate oxygen 1) forms one stable hydrogen bond with G453 and the adjacent OP2 forms approximately two (1.86) salt bridges with R489, the latter being located at the beginning of the NKR finger. Overall, the binding pocket of the mC-C’ simulation shows a regular and stable polar interaction pattern. This pattern is nearly identical to the one observed in the mC-mC’ and mC-caC’ simulations (Fig 4c and 4e), indicating that modifications on the distal strand have little effect on the conformation and interactions of the nucleotide binding pocket containing flipped mC.

Analysis of the binding mode of the hemi-modified caC-C’ system (Fig 4b) shows that this modification leads to a very different interaction pattern: The previously observed hydrogen bonds of the nucleotide N4 atom are substantially weakened (-1.87 hydrogen bonds), while interactions of O2 are dispersed from two to three amino acids (-0.2 hydrogen bonds total). Although several hydrogen bond donors such as S486, N509, and the backbone atoms of I454 and G453 are available in the binding pocket, the carboxyl atoms O51 and O52 of caC predominantly interact with R489, forming very strong interactions (1.92 salt bridges on average) with this residue. This interaction pattern is unexpected, since the caC modification is located within the binding site, whereas R489 is located at its edge, usually interacting only with the DNA backbone. This may cause a force pulling the base out of position and could explain the weaker hydrogen bonds formed by the base’s N4 nitrogen. The NKR finger region consisting of residues 488 to 502 is a flexible loop important for DNA binding with residues N494, K495, and R496 at its tip. Observing that R489 is involved directly in interactions with the carboxyl oxygens establishes a direct link between the flipped-out base and the NKR finger, which predominantly interacts with the distal DNA strand. The interaction pattern of the caC-caC’ system (Fig 4d) is consistent with this observation. In this system, the caC N4 and O2 atoms show an overall similar interaction pattern to the hemi-modified variant. However, distinct differences are seen in the interaction with R489: The salt bridges between the carboxyl oxygens and R489 are much weaker (only 0.5), whereas the residue forms very strong interactions (3.03) with the backbone atoms OP1 and OP2 (+ 0.96 compared to mC-C’). To compensate for the weaker R489 interactions, O51 and O52 form fluctuating weak (≤ 0.5) hydrogen bonds with S486 and G453 in the binding pocket. The caC-mC’ system (Fig 4f) shows a mixture between these patterns, as R489 establishes 1.17 salt bridges to O51 and O52 of caC and 2.64 salt bridges to the caC backbone. The hydrogen bonds of the carboxyl oxygens are more dispersed compared to the caC-caC’ system, interacting weakly (< 0.5) with S486, N509, and I454 and moderately strong (0.74) with G453. In turn, O2 establishes only 0.7 hydrogen bonds to G470, G469, and A468, which is 1 less than in caC-caC’. The differences we observed in the binding modes of caC-C’, caC-caC’ and caC-mC’ indicate that the caC carboxyl oxygens have several possible interaction partners in the nucleotide binding pocket and the interaction networks are more heterogenous compared to bound mC. In addition to interactions within the binding pocket (S486, N509, I454, G453), caC oxygens O51/O52 can establish alternative interactions outside of the main pocket, particularly with the NKR finger residue R489. In combination with our observation that the overall interaction pattern of R489 is strongly dependent on the xC’ modification on the distal strand, this suggests that the binding mode is influenced by the NKR finger, which senses that modification.

Another notable difference between the interaction networks is the hydrogen bond of the Y471 hydroxyl atom to the OP2 atom of the modified base, which is absent in the carboxylated variants (Fig 4). As Y471 has been described previously to form a hydrophobic cage, closing like a lid over the modified base [2], we analysed whether the distances between the tyrosine and pyrimidine rings were influenced by the nucleotide modification. S8 Fig shows that for both mC-C’ and mC-mC’ the distances cluster in two close narrow peaks with tyrosine being stabilized in its position, while for the carboxylated variants the distances fluctuate between multiple distinct conformations due to changes in the nucleotide binding mode. The distance histograms tend to differ more between replicas than during a single simulation, indicating that Y471 flips between distinct conformations with characteristic transition times roughly in the ~ 10–100 ns range or longer. Interestingly, the distribution of mC-caC’ shows a similar pattern to the other methylated variants, but an additional small peak at 8–9 Å, indicating a partial destabilization of the Y471 lid. In summary, carboxylation of the flipped base leads to a different local conformation of the binding pocket compared to methylation. While during the simulations of complexes featuring a flipped mC base very similar binding modes were observed, strong differences were found in the binding modes of complexes containing a flipped caC depending on the xC’ modification on the distal strand. These differences suggest potential conformational long-range correlations between the binding pocket and the NKR finger, in particular R489, which can interact directly with the carboxyl modification of the flipped-out base.

#### Analysis of mC and caC recognition on the distal DNA strand by the UHRF1-SRA NKR finger

**Fig 5:**
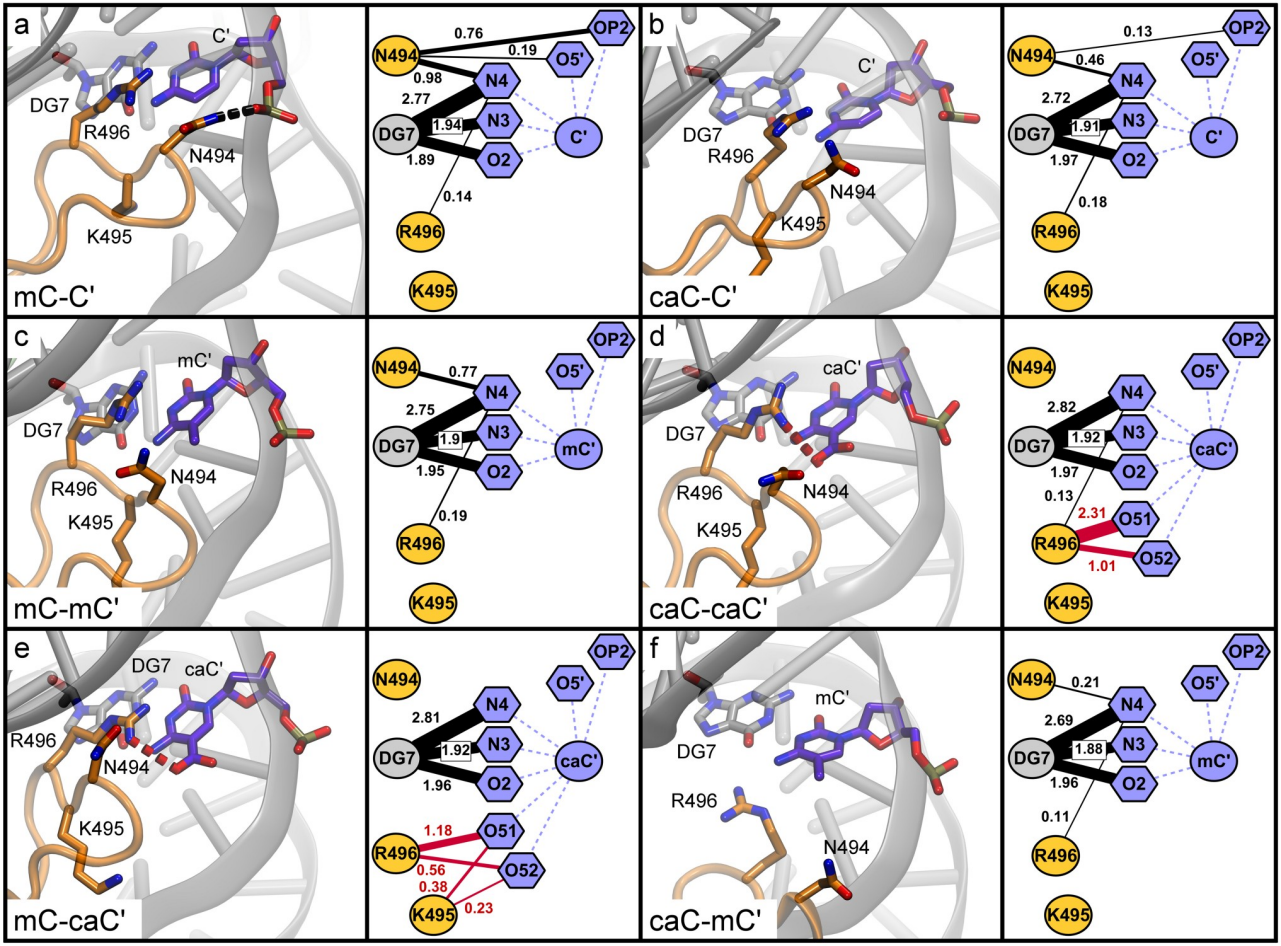
Interaction networks of the NKR finger based on molecular dynamics simulations of UHRF1-SRA. Structures show representative conformations of the NKR finger close to the distal (symmetrical) DNA modification site as observed during the MD simulations. To the right of each structure a corresponding network of hydrogen bonds (black lines) and salt bridges (red lines) over the course of the simulation is shown. Numbers next to edges show the average number of interactions per time frame. Edges representing interactions occurring in ≤ 10% of simulation time are omitted for clarity. For node pairs featuring both hydrogen bonds and salt bridges, only salt bridges are displayed.

Our observations so far indicated that the NKR finger could play an important role for UHRF1 to differentiate between carboxylated and methylated CpG sites. As for the binding pocket, we analysed the interaction networks between the finger residues and the second modification site on the distal DNA strand (Fig 5). In the native binding conformation represented by the mC-C’ simulation (Fig 5a), N494 forms 0.76 hydrogen bonds with the OP2 atom of the unmodified DNA base backbone. This interaction has been described previously as one of the key features for differentiating between hemi-methylated and symmetrically methylated DNA [29, 33]. This is in line with our simulation of mC-mC’ in which this interaction is not observed (Fig 5c), as N494 is pushed away from its native position by steric repulsion of the additional methyl group. Interestingly, a similar trend is observed for caC-C’ (Fig 5b), for which the N494-OP2 hydrogen bond is also much weaker (0.13) compared to mC-C' despite the lack of any modification on the distal DNA strand. This indicates a shift in the conformation of the NKR finger similar to the mC-mC’ system, only that in this case the cause is not the modified base on the distal strand, but it appears that the shift might be mediated by the conformations of R489 as described above. Investigating the interaction pattern of the caC-caC’ system (Fig 5d), we observed additional strong salt bridges (3.32) between R496 and the caC’ O51/O52 atoms. No interactions are formed between the modified base and N494, likely related to steric repulsion similar to the methyl group as in mC-mC'. The interaction pattern of mC-caC’ (Fig 5e) is similar to caC-caC’, but with slightly weaker individual interactions as R496 forms only 1.74 salt bridges to the carboxyl oxygens (- 1.58), albeit with support from spurious interactions of K495 (0.61). In contrast, the interaction pattern of caC-mC’ (Fig 5f) resembles mC-mC’ with an additional loss of 0.51 hydrogen bonds between N494 and the N4 base atom of mC’, with nearly no polar interactions remaining between the NKR finger and the modified base.

**Fig 6:**
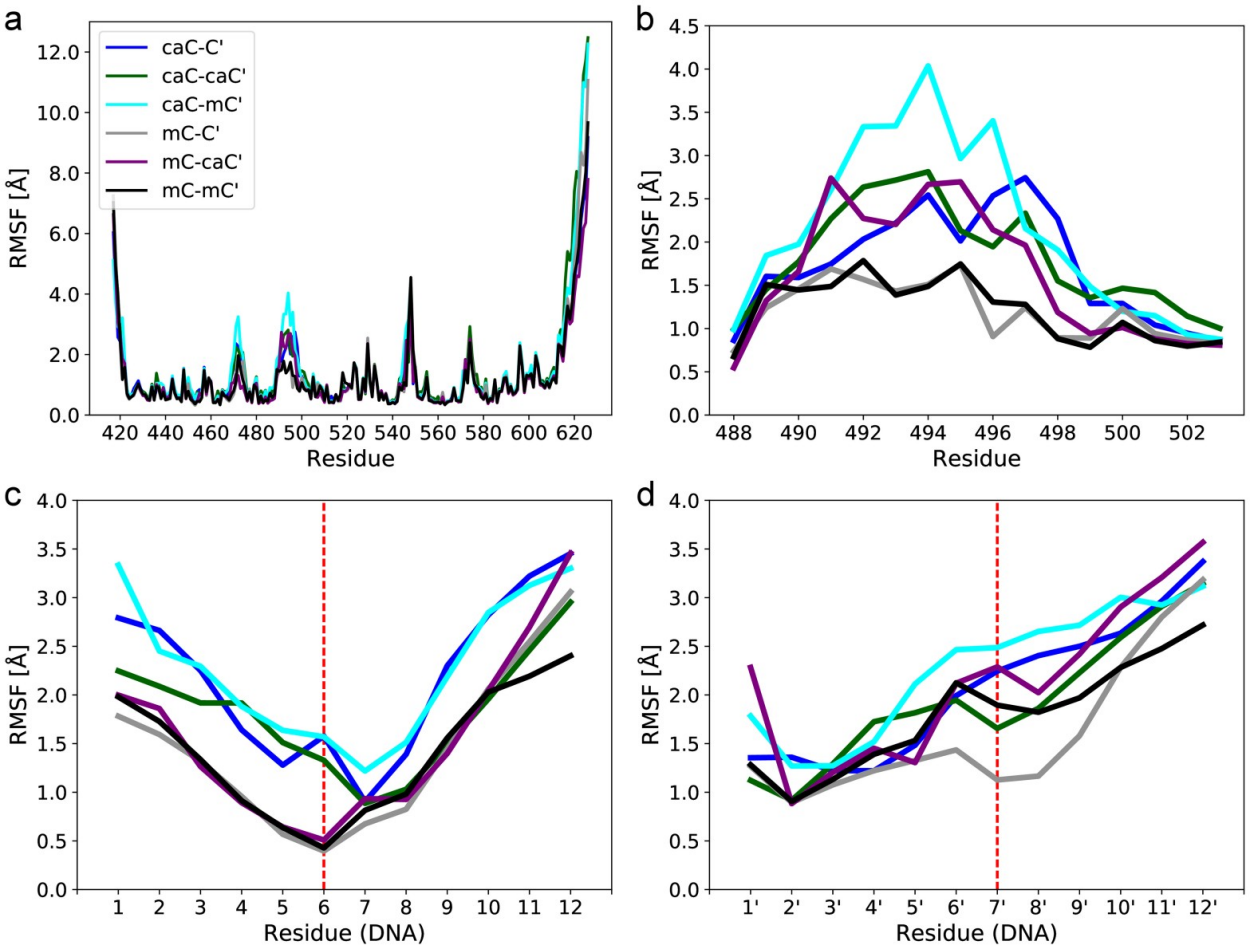
Root Mean Square Fluctuation (RMSF) of protein and DNA regions in molecular dynamics trajectories of UHRF1-SRA. (a) Full protein. (b) NKR finger. (c) DNA strand containing the flipped xC base bound by the protein. (d) Distal DNA strand containing the modified xC’ base. Red dashed lines show the xC/xC’ modification sites.

R496 is generally a strong interaction partner for the DNA in all simulated systems, partaking in hydrogen bonds with adjacent bases and stacking interactions with the modified base. The interactions of the carboxyl group seem to modulate this role, either directly through salt bridges or by influencing stacking, although stacking effects are not quantifiable using classical force fields. As our analyses showed that only mC-C’ retained the native interaction pattern of the NKR finger, we were interested in whether there was any effect on the flexibility of the finger. To quantify this, we compared the Root Mean Square Fluctuation (RMSF) for all protein residues (Fig 6a). Overall, very similar residue flexibility is observed for most regions of the protein independent of DNA modifications. Only two regions show substantial differences: The first is located in the region between residues 468 and 475, which corresponds to the conformational flexibility of Y471 discussed above. The second region featuring pronounced differences is located between residues 488 and 502 forming the NKR finger (Fig 6b). Although the NKR finger shows a different conformation in the mC-mC’ simulation, the flexibility of the finger is comparable to the mC-C’ reference system. In contrast, for the caC-C’, caC-caC’, and mC-caC’ systems, the finger shows increased flexibility with a slightly different pattern: The hemi-modified variant being more flexible in the 495–499 region and both the caC-caC’ and mC-caC’ variants more flexible between residues 490 and 494. Finally, the largest finger flexibility of all systems is observed for caC-mC’, in line with the previously observed loss of interactions of the NKR finger.

UHRF1 encloses the flipped base by inserting a thumb into the minor groove and the NKR finger into the major groove of the DNA strands. Having observed differences in interaction pattern and flexibility of the NKR finger depending on the CpG modification pattern, we asked how the DNA structure around the modified sites was affected. Fig 6c shows that overall flexibility of the bound strand increases if caC is in the binding pocket, including particularly strong differences at the flipped xC base in position 6. For the distal strand, flexibility compared to mC-C’ increases in all systems around the modified base 7’ (Fig 6d), likely reflecting the loss of the stabilizing hydrogen bond between N494 and the DNA backbone. For a more detailed analysis, we examined how the modified bases affected the minor and major grooves, as they are strongly influenced by shifts in the DNA backbone. A small but consistent increase of minor groove width by about 1–2 Å is observed between base pairs 3 to 5 in all simulations containing caC in the binding pocket, while widths decrease by roughly the same amount between base pairs 7 and 9 (S9 Fig; locations of base pairs are shown in Fig 1d). The major groove follows a similar but weaker trend due to the large variances within replicas (S10 Fig). Although individual effects are small, their consistency and anti-symmetry with respect to the modified bases 6 and 7’ is notable. Therefore, the flipped base appears to be important for the local flexibility of the DNA backbone, which is more rigid for mC and more flexible for caC. This could potentially contribute to the increased flexibility of NKR finger residues, particularly R489, which is in a prime position to sense distortions due to its strong salt bridges with the phosphate backbone of the flipped base. These observations agree with our interaction network analyses, showing that binding of a flipped caC base leads to conformational rearrangements including the DNA strands in locations close to the modification sites.

In summary, our simulations reveal that all DNA modifications investigated lead to differences in the conformation and binding pattern of the nucleotide binding pocket and NKR finger compared to the native conformation of the mC-C’ system. Interestingly, in the hemi-carboxylated variant caC-C’, local conformational changes in the binding pocket are transmitted to the NKR finger via R489, which in turn becomes more flexible and thus compromises the essential N494 hydrogen bond to the C’ backbone on the distal strand [29]. The symmetrically carboxylated variant caC-caC’ also shows increased NKR finger flexibility, but different interaction patterns, particularly for R489 and R496. The latter forms strong salt bridges with the caC’ modified base, possibly compensating for the loss of the N494 hydrogen bond. This is in strong contrast to the recognition of hemi- and symmetrically methylated CpG sites, which show much smaller differences. Our additional analysis of the hybrid modification variant mC-caC’ suggests that the NKR finger can recognize and interact with the caC’ modification without large changes in the binding pocket containing a flipped mC. In the opposite case of caC-mC’, a heterogeneous binding pocket conformation is met with an almost complete loss of NKR finger interactions with the mC’ base. Based on this simulation data, we formulate the hypothesis that UHRF1 binding of a flipped-out caC base leads to conformational changes in the protein, which can propagate to and induce shifts in the protein’s NKR finger and the DNA backbone. In turn, modification of the distal DNA strand can influence the overall binding mode via steric repulsion or attractive interactions with the NKR finger, coupling recognition of both modification sites.

## Discussion

The role of UHRF1 as a specific hemi-mC reader is well established [1, 3]. Reported dissociation constants range from 1.8 nM to 9.23 μM depending on the protein construct and DNA substrate [1, 32, 36, 59, 60] (Table 1). Here, we use a relatively long DNA fragment (42 bp) with a single modified CpG site, whereas other studies have used either oligonucleotides with multiple methylated sites [1] or shorter DNA fragments with one modification site [29, 32]. We observe a relatively low preference of hemi-methylated over unmodified DNA compared to published data [1, 19, 29, 32], which we explain by the lower density of methylated sites in our experiments. To verify this relation, we also measured binding of a shorter DNA fragment which increased the affinity of UHRF1 for hemi-mC to the order of what has been reported in literature [32, 60]. A possible explanation can be given by the proposed “sliding” mechanism of UHRF1 [60, 61, 68, 69]: In this model, fast unspecific binding occurs between the protein and DNA, followed by a sliding “scan” for a modified base. Thus, the relative differences in apparent binding affinities would decrease with the length of the DNA fragments, which corresponds to our observations. In three independent assays, we observe that UHRF1 prefers binding symmetrically carboxylated CpG sites over the hemi-carboxylated variant, which is the opposite behaviour as observed for methylcytosine. Interestingly, we also measure increased affinity of UHRF1 towards hybrid mC-caC’ sites. To find a possible explanation for the underlying molecular mechanisms of these differences, we performed MD simulations of the UHRF1-SRA domain in complex with hemi-, hybrid, and symmetrically modified DNA based on the crystal structure of mC-C’, which features the flipped-out base in the protein’s binding pocket and the second potentially modified base on the distal strand in the flipped-in state. As discussed in the results section, we preferred this approach over simulating the entire flipping process.

Our simulations revealed substantial differences in the conformations and binding patterns of the nucleotide binding pocket and the NKR finger between caC and mC modifications. If caC is bound in the binding pocket, these two regions appear to be coupled and able to influence each other in a more pronounced manner than for mC. In the caC-C’ system, this coupling leads to reduced hydrogen bonding between N494 and the DNA backbone, which is an essential interaction for binding [29]. The same interaction is interrupted by steric repulsion when mC’ and caC’ modifications are present on the distal strand, sterically pushing the NKR finger out of its native binding position. The simulations provide no indication that the mC’ modification could be beneficial to overall binding, but the caC’ modification forms stable salt bridges to the NKR finger, which might compensate for the loss of the N494-DNA hydrogen bond. Thus, the caC’ oxygens push the NKR finger away from its hydrogen bond with the DNA backbone and at the same time offer salt bridges to bind the finger in its new position. In this light, we propose that the carboxyl group of both, the caC and caC’ bases, has a strong influence on their local interaction network partners in UHRF1, leading to conformational changes in which R489, N494, and R496 play key roles in differentiating DNA modifications. Other proteins are already known to recognize caC’ modifications using finger regions: TET3, one of the three dioxygenases that generate hmC, fC, and caC, was also shown to specifically bind symmetrically carboxylated CpG sites with a finger-like structure containing a NRRT sequence [23]. Comparing the NKRT sequence of UHRF1 to the NRRT sequence of TET3, it is intriguing to speculate that such a flexible stretch of basic amino acids facilitates the binding of distant carboxyl groups.

The biological role of UHRF1 binding to symmetrically carboxylated DNA remains to be determined, considering the low abundance of this modification in cells. For this reason, it is likely that the majority of UHRF1 in a proliferating cell population interacts with hemi-methylated CpG sites, but a certain fraction may encounter and bind mC-caC’ and caC-caC’ depending on the cell type and cell cycle phase. Carboxylcytosine has been suggested to be an intermediate of active DNA demethylation and is detected at gene regulatory elements and promoters of actively transcribed genes, indicating dynamic DNA methylation turnover [14–16]. Several DNA repair mechanisms have been associated with this demethylation [70–72], most prominently removal of fC and caC by TDG and the base excision repair pathway [8, 73–75]. Interestingly, both UHRF1 and UHRF2 have been shown to play a role in DNA damage response [76–78]. Additionally, the bona fide UHRF1 interaction partner DNMT1 has been described to change its genomic localization upon oxidative stress [79, 80]. Furthermore, besides being demethylation intermediates, fC and caC are thought to influence DNA replication and genome stability [81, 82]. By transiently pausing RNA polymerases, fC and caC may lead to precise fine-tuning of gene expression [21]. Accordingly, the binding of UHRF1 to caC as demonstrated in our study could also represent a way of locus-specific gene expression regulation in addition to its well-established role in recognizing hemi-mC sites and initiating DNA maintenance methylation. Last but not least, UHRF1 has recently been described as a regulator of bivalent promoters and an interactor of SETD1A [83]. Interestingly, both functions have been attributed to TET proteins as well [84, 85]. This raises the intriguing possibility that UHRF1 integrates several epigenetic marks at bivalent domains and that caC, generated by TET proteins, is one of these marks involved in maintenance of the bivalent state. However, further work is needed to determine whether and where exactly UHRF1 binds caC sites in vivo and what implications this might have on epigenetic gene regulation.

## Supporting information

S1 Fig Raw gel images of EMSA experiments. All raw gel scans that have been used to generate the EMSA results presented in Fig 2b/2c and S5 Fig. An overview of all individual quantitative values and the corresponding statistics is provided on page 1.
(PDF)

S2 Fig Normalized MST traces of UHRF1 bound to C-C’, mC-C’, mC-caC’ and caC-caC’. Fluorescence traces that have been used to generate the binding curves in Fig 3. Traces are shown individually for all modifications and are coloured by experimental replicate. Blue and red bars indicate the time points that were used for the analysis; blue: t_cold_ (pre infra-red laser), red: t_hot_ (post infra-red laser).
(TIF)

S3 Fig Root Mean Squared Deviation (RMSD) of DNA atoms in molecular dynamics trajectories of UHRF1-SRA. Coordinates were fitted to the initial crystal structure using the Cα atoms of protein residues 432 to 586. Only the last 1000 frames of each trajectory were used for analysis (vertical lines). Horizontal lines were added at 4 Å to highlight trajectories with strong structural distortions.
(TIF)

S4 Fig Root Mean Squared Deviation (RMSD) of protein atoms in molecular dynamics trajectories of UHRF1-SRA. Coordinates were fitted to the initial crystal structure using the Cα atoms of protein residues 432 to 586. Only the last 1000 frames of each trajectory were used for analysis (vertical lines). Horizontal lines were added at 4 Å to highlight trajectories with strong structural distortions.
(TIF)

S5 Fig EMSAs of UHRF2 with differentially modified DNA. Quantitation of the bound fraction of EMSAs of wild type UHRF2-GFP with 42 bp DNA oligonucleotides carrying different cytosine modifications. Experiments and analyses have been performed as in Fig 2.
(TIF)

S6 Fig Melting temperatures of modified DNA in presence of UHRF1-SRA. (a) The melting temperature of double-stranded DNA containing C-C’ in a CpG context (red) or no CpG site (black) with (solid lines) or without (dotted lines) a 5-fold excess of the SRA domain of UHRF1, measured using high resolution melting temperature (HRM) analysis. As control, proteins were digested by proteinase K before HRM analysis (right panel). Experiments were performed independently three times; one representative experiment is depicted as average of three technical replicates. (b) Melting temperatures as in (a) with DNA harbouring symmetric caC (green) or hemi-mC (gray) at the central CpG site.
(TIF)

S7 Fig Size exclusion chromatograms of differentially modified DNA in the presence or absence of UHRF1-SRA. To test for different binding stoichiometries of the SRA domain towards differentially modified DNA, ATTO550-labeled DNA oligonucleotides were incubated with a 10-fold excess of SRA. Size exclusion chromatograms of analyzed DNA oligonucleotides at an absorbance of 554 nm (a) and 260nm/280nm (b) show a clear and comparable shift in retention time for the SRA-bound DNA (left peaks) compared to free DNA (right peaks).
(TIF)

S8 Fig Histograms of distances between Y471 and the flipped-out DNA base in molecular dynamics trajectories of UHRF1-SRA. Individual replicas are shown as separate bars stacked on top of each other. Distances were measured between the geometric centres of the phenyl and pyrimidine rings. Red lines show a gaussian kernel estimate of the probability density function (pdf). The estimated pdf of the mC-C’ system is shown as black dashed lines.
(TIF)

S9 Fig Distribution of DNA minor groove widths in molecular dynamics trajectories of UHRF1-SRA. Blue faces represent gaussian kernel estimates of the underlying values. Black bars show distribution means and standard deviations.
(TIF)

S10 Fig Distribution of DNA major groove widths in molecular dynamics trajectories of UHRF1-SRA. Blue faces represent gaussian kernel estimates of the underlying values. Black bars show distribution means and standard deviations.
(TIF)

S1 Text Additional experimental procedures. (DOCX)

S1 File Parameter files for mC/caC used during molecular dynamics simulations. (ZIP)

## References

[1] Bostick M, Kim JK, Esteve P-O, Clark A, Pradhan S, Jacobsen SE. UHRF1 Plays a Role in Maintaining DNA Methylation in Mammalian Cells. Science. 2007;317:1760–4. 10.1126/science.1147939 . 17673620

[2] Hashimoto H, Horton JR, Zhang X, Bostick M, Jacobsen SE, Cheng X. The SRA domain of UHRF1 flips 5-methylcytosine out of the DNA helix. Nature. 2008;455:826–9. 10.1038/nature07280 . 18772888 PMC2602803

[3] Sharif J, Muto M, Takebayashi S, Suetake I, Iwamatsu A, Endo TA, et al The SRA protein Np95 mediates epigenetic inheritance by recruiting Dnmt1 to methylated DNA. Nature. 2007;450(7171):908–12. 10.1038/nature06397 . 17994007

[4] Nishiyama A, Yamaguchi L, Sharif J, Johmura Y, Kawamura T, Nakanishi K, et al Uhrf1-dependent H3K23 ubiquitylation couples maintenance DNA methylation and replication. Nature. 2013;502(7470):249–53. 10.1038/nature12488 . 24013172

[5] Qin W, Wolf P, Liu N, Link S, Smets M, La Mastra F, et al DNA methylation requires a DNMT1 ubiquitin interacting motif (UIM) and histone ubiquitination. Cell Res. 2015;25(8):911–29. 10.1038/cr.2015.72 . 26065575 PMC4528052

[6] Ishiyama S, Nishiyama A, Saeki Y, Moritsugu K, Morimoto D, Yamaguchi L, et al Structure of the Dnmt1 Reader Module Complexed with a Unique Two-Mono-Ubiquitin Mark on Histone H3 Reveals the Basis for DNA Methylation Maintenance. Mol Cell. 2017;68(2):350–60 e7. 10.1016/j.molcel.2017.09.037 . 29053958

[7] Tahiliani M, Koh KP, Shen Y, Pastor WA, Bandukwala H, Brudno Y, et al Conversion of 5-methylcytosine to 5-hydroxymethylcytosine in mammalian DNA by MLL partner TET1. Science. 2009;324(5929):930–5. 10.1126/science.1170116 . 19372391 PMC2715015

[8] He YF, Li BZ, Li Z, Liu P, Wang Y, Tang Q, et al Tet-mediated formation of 5-carboxylcytosine and its excision by TDG in mammalian DNA. Science. 2011;333(6047):1303–7. 10.1126/science.1210944 . 21817016 PMC3462231

[9] Pfaffeneder T, Hackner B, Truss M, Munzel M, Muller M, Deiml CA, et al The discovery of 5-formylcytosine in embryonic stem cell DNA. Angew Chem Int Ed Engl. 2011;50(31):7008–12. 10.1002/anie.201103899 . 21721093

[10] Nabel CS, Kohli RM. Molecular biology. Demystifying DNA demethylation. Science. 2011;333(6047):1229–30. 10.1126/science.1211917 . 21885763

[11] Pfaffeneder T, Spada F, Wagner M, Brandmayr C, Laube SK, Eisen D, et al Tet oxidizes thymine to 5-hydroxymethyluracil in mouse embryonic stem cell DNA. Nat Chem Biol. 2014;10(7):574–81. 10.1038/nchembio.1532 . 24838012

[12] Globisch D, Münzel M, Müller M, Michalakis S, Wagner M, Koch S, et al Tissue distribution of 5-hydroxymethylcytosine and search for active demethylation intermediates. PLoS ONE. 2010;5:1–9. 10.1371/journal.pone.0015367 . 21203455 PMC3009720

[13] Eleftheriou M, Pascual AJ, Wheldon LM, Perry C, Abakir A, Arora A, et al 5-Carboxylcytosine levels are elevated in human breast cancers and gliomas. Clinical epigenetics. 2015;7 10.1186/s13148-015-0117-x . 26300993 PMC4546187

[14] Lu X, Han D, Boxuan Simen Z, Song C-X, Zhang L-S, Doré LC, et al Base-resolution maps of 5-formylcytosine and 5-carboxylcytosine reveal genome-wide DNA demethylation dynamics. Cell Research. 2015;25:386–9. 10.1038/cr.2015.5 . 25591929 PMC4349244

[15] Neri F, Incarnato D, Krepelova A, Rapelli S, Anselmi F, Parlato C, et al Single-Base resolution analysis of 5-formyl and 5-carboxyl cytosine reveals promoter DNA Methylation Dynamics. Cell Reports. 2015;10:674–83. 10.1016/j.celrep.2015.01.008 . 25660018

[16] Shen L, Wu H, Diep D, Yamaguchi S, D'Alessio AC, Fung HL, et al Genome-wide analysis reveals TET- and TDG-dependent 5-methylcytosine oxidation dynamics. Cell. 2013;153:692–706. 10.1016/j.cell.2013.04.002 . 23602152 PMC3687516

[17] Bronner C, Achour M, Arima Y, Chataigneau T, Saya H, Schini-Kerth VB. The UHRF family: oncogenes that are drugable targets for cancer therapy in the near future? Pharmacol Ther. 2007;115(3):419–34. 10.1016/j.pharmthera.2007.06.003 . 17658611

[18] Pichler G, Wolf P, Schmidt CS, Meilinger D, Schneider K, Frauer C, et al Cooperative DNA and histone binding by Uhrf2 links the two major repressive epigenetic pathways. J Cell Biochem. 2011;112(9):2585–93. 10.1002/jcb.23185 . 21598301 PMC3569875

[19] Spruijt CG, Gnerlich F, Smits AH, Pfaffeneder T, Jansen PWTC, Bauer C, et al Dynamic readers for 5-(Hydroxy)methylcytosine and its oxidized derivatives. Cell. 2013;152:1146–59. 10.1016/j.cell.2013.02.004 . 23434322

[20] Rajakumara E, Nakarakanti NK, Nivya MA, Satish M. Mechanistic insights into the recognition of 5-methylcytosine oxidation derivatives by the SUVH5 SRA domain. Scientific Reports. 2016;6:20161 10.1038/srep20161 26841909 PMC4740795

[21] Wang L, Zhou Y, Xu L, Xiao R, Lu X, Chen L, et al Molecular basis for 5-carboxycytosine recognition by RNA polymerase II elongation complex. Nature. 2015;523:621–5. 10.1038/nature14482 . 26123024 PMC4521995

[22] Hashimoto H, Olanrewaju YO, Zheng Y, Wilson GG, Zhang X, Cheng X. Wilms tumor protein recognizes 5-carboxylcytosine within a specific DNA sequence. Genes Dev. 2014;28(20):2304–13. 10.1101/gad.250746.114 . 25258363 PMC4201290

[23] Jin S-G, Zhang Z-M, Dunwell TL, Harter MR, Wu X, Johnson J, et al Tet3 reads 5-carboxylcytosine through its CXXC domain and is a potential guardian against neurodegeneration. Cell Rep. 2016;14:493–505. 10.1016/j.celrep.2015.12.044 . 26774490 PMC4731272

[24] Gowher H, Jeltsch A. Mammalian DNA methyltransferases: new discoveries and open questions. Biochemical Society Transactions. 2018;46(5):1191–202. 10.1042/BST20170574 30154093 PMC6581191

[25] Arand J, Spieler D, Karius T, Branco MR, Meilinger D, Meissner A, et al In vivo control of CpG and non-CpG DNA methylation by DNA methyltransferases. PLoS Genet. 2012;8(6):e1002750 10.1371/journal.pgen.1002750 . 22761581 PMC3386304

[26] Xu L, Chen YC, Chong J, Fin A, McCoy LS, Xu J, et al Pyrene-based quantitative detection of the 5-formylcytosine loci symmetry in the CpG duplex content during TET-dependent demethylation. Angew Chem Int Ed Engl. 2014;53(42):11223–7. 10.1002/anie.201406220 . 25159856 PMC4227401

[27] Yu M, Hon GC, Szulwach KE, Song CX, Zhang L, Kim A, et al Base-resolution analysis of 5-hydroxymethylcytosine in the mammalian genome. Cell. 2012;149(6):1368–80. 10.1016/j.cell.2012.04.027 . 22608086 PMC3589129

[28] Arita K, Ariyoshi M, Tochio H, Nakamura Y, Shirakawa M. Recognition of hemi-methylated DNA by the SRA protein UHRF1 by a base-flipping mechanism. Nature. 2008;455(7214):818–21. 10.1038/nature07249 . 18772891

[29] Avvakumov GV, Walker JR, Xue S, Li Y, Duan S, Bronner C, et al Structural basis for recognition of hemi-methylated DNA by the SRA domain of human UHRF1. Nature. 2008;455(7214):822–5. 10.1038/nature07273 . 18772889

[30] Frauer C, Hoffmann T, Bultmann S, Casa V, Cardoso MC, Antes I, et al Recognition of 5-hydroxymethylcytosine by the Uhrf1 SRA domain. PLoS ONE. 2011;6:1–8. 10.1371/journal.pone.0021306 . 21731699 PMC3120858

[31] Hashimoto H, Liu Y, Upadhyay AK, Chang Y, Howerton SB, Vertino PM, et al Recognition and potential mechanisms for replication and erasure of cytosine hydroxymethylation. Nucleic Acids Research. 2012;40(11):4841–9. 10.1093/nar/gks155 22362737 PMC3367191

[32] Zhou T, Xiong J, Wang M, Yang N, Wong J, Zhu B, et al Structural Basis for Hydroxymethylcytosine Recognition by the SRA Domain of UHRF2. Molecular Cell. 2014;54:879–86. 10.1016/j.molcel.2014.04.003 . 24813944

[33] Bianchi C, Zangi R. UHRF1 discriminates against binding to fully-methylated CpG-Sites by steric repulsion. Biophysical Chemistry. 2013;171:38–45. 10.1016/j.bpc.2012.10.002 . 23245651

[34] Hashimoto H, Zhang X, Cheng X. Activity and crystal structure of human thymine DNA glycosylase mutant N140A with 5-carboxylcytosine DNA at low pH. DNA Repair. 2013;12:535–40. 10.1016/j.dnarep.2013.04.003 . 23680598 PMC3758246

[35] Gelato KA, Tauber M, Ong MS, Winter S, Hiragami-Hamada K, Sindlinger J, et al Accessibility of different histone H3-binding domains of UHRF1 is allosterically regulated by phosphatidylinositol 5-phosphate. Mol Cell. 2014;54(6):905–19. 10.1016/j.molcel.2014.04.004 . 24813945

[36] Fang J, Cheng J, Wang J, Zhang Q, Liu M, Gong R, et al Hemi-methylated DNA opens a closed conformation of UHRF1 to facilitate its histone recognition. Nat Commun. 2016;7:11197 10.1038/ncomms11197 . 27045799 PMC4822050

[37] Harrison JS, Cornett EM, Goldfarb D, DaRosa PA, Li ZM, Yan F, et al Hemi-methylated DNA regulates DNA methylation inheritance through allosteric activation of H3 ubiquitylation by UHRF1. Elife. 2016;5 10.7554/eLife.17101 . 27595565 PMC5012860

[38] Vaughan RM, Dickson BM, Whelihan MF, Johnstone AL, Cornett EM, Cheek MA, et al Chromatin structure and its chemical modifications regulate the ubiquitin ligase substrate selectivity of UHRF1. Proc Natl Acad Sci U S A. 2018;115(35):8775–80. 10.1073/pnas.1806373115 . 30104358 PMC6126761

[39] Rottach A, Frauer C, Pichler G, Bonapace IM, Spada F, Leonhardt H. The multi-domain protein Np95 connects DNA methylation and histone modification. Nucleic Acids Res. 2010;38(6):1796–804. 10.1093/nar/gkp1152 . 20026581 PMC2847221

[40] Ivani I, Dans PD, Noy A, Perez A, Faustino I, Hospital A, et al Parmbsc1: a refined force field for DNA simulations. Nat Methods. 2016;13(1):55–8. 10.1038/nmeth.3658 . 26569599 PMC4700514

[41] Lankaš F, Cheatham TE, Špačáková Na, Hobza P, Langowski J, Šponer J. Critical Effect of the N2 Amino Group on Structure, Dynamics, and Elasticity of DNA Polypurine Tracts. Biophysical Journal. 2002;82(5):2592–609. 10.1016/s0006-3495(02)75601-4 11964246 PMC1302048

[42] Perez A, Marchan I, Svozil D, Sponer J, Cheatham TE 3rd, Laughton CA, et al Refinement of the AMBER force field for nucleic acids: improving the description of alpha/gamma conformers. Biophys J. 2007;92(11):3817–29. 10.1529/biophysj.106.097782 . 17351000 PMC1868997

[43] Cieplak P, Cornell Wendy D, Bayly C, Kollman Peter A. Application of the multimolecule and multiconformational RESP methodology to biopolymers: Charge derivation for DNA, RNA, and proteins. Journal of Computational Chemistry. 1995;16(11):1357–77. 10.1002/jcc.540161106

[44] Vanquelef E, Simon S, Marquant G, Garcia E, Klimerak G, Delepine JC, et al R.E.D. Server: a web service for deriving RESP and ESP charges and building force field libraries for new molecules and molecular fragments. Nucleic Acids Res. 2011;39(Web Server issue):W511–7. 10.1093/nar/gkr288 . 21609950 PMC3125739

[45] Wang F, Becker J-P, Cieplak P, Dupradeau F-Y. R.E.D. Python: Object oriented programming for Amber force fields. Université de Picardie—Jules Verne, Sanford Burnham Prebys Medical Discovery Institute. 2013.

[46] Dupradeau FY, Pigache A, Zaffran T, Savineau C, Lelong R, Grivel N, et al The R.E.D. tools: advances in RESP and ESP charge derivation and force field library building. Phys Chem Chem Phys. 2010;12(28):7821–39. 10.1039/c0cp00111b . 20574571 PMC2918240

[47] Bayly CI, Cieplak P, Cornell W, Kollman PA. A well-behaved electrostatic potential based method using charge restraints for deriving atomic charges: the RESP model. The Journal of Physical Chemistry. 1993;97(40):10269–80. 10.1021/j100142a004

[48] Frisch MJ, Trucks GW, Schlegel HB, Scuseria GE, Robb MA, Cheeseman JR, et al. Gaussian 09 Revision A.2. 2009.

[49] Case DA, Cerutti DS, T.E. Cheatham I, Darden TA, Duke RE, Giese TJ, et al. AMBER 2017. University of California, San Francisco. 2017.

[50] Maier JA, Martinez C, Kasavajhala K, Wickstrom L, Hauser KE, Simmerling C. ff14SB: Improving the Accuracy of Protein Side Chain and Backbone Parameters from ff99SB. J Chem Theory Comput. 2015;11(8):3696–713. 10.1021/acs.jctc.5b00255 . 26574453 PMC4821407

[51] Jorgensen WL, Chandrasekhar J, Madura JD, Impey RW, Klein ML. Comparison of simple potential functions for simulating liquid water. The Journal of Chemical Physics. 1983;79(2):926 10.1063/1.445869

[52] Miyamoto S, Kollman PA. Settle—an Analytical Version of the Shake and Rattle Algorithm for Rigid Water Models. Journal of Computational Chemistry. 1992;13(8):952–62.

[53] Duell ER, Glaser M, Le Chapelain C, Antes I, Groll M, Huber EM. Sequential Inactivation of Gliotoxin by the S-Methyltransferase TmtA. ACS chemical biology. 2016;11(4):1082–9. 10.1021/acschembio.5b00905 . 26808594

[54] Roe DR, Cheatham TE 3rd. PTRAJ and CPPTRAJ: Software for Processing and Analysis of Molecular Dynamics Trajectory Data. J Chem Theory Comput. 2013;9(7):3084–95. 10.1021/ct400341p . 26583988

[55] El Hassan MA, Calladine CR. Two distinct modes of protein-induced bending in DNA. J Mol Biol. 1998;282(2):331–43. 10.1006/jmbi.1998.1994 9735291

[56] Humphrey W, Dalke A, Schulten K. VMD: visual molecular dynamics. J Mol Graph. 1996;14(1):33–8, 27–8. 10.1016/0263-7855(96)00018-5 . 8744570

[57] Hunter JD. Matplotlib: A 2D Graphics Environment. Computing in Science & Engineering. 2007;9(3):90–5. 10.1109/MCSE.2007.55

[58] Seidel SA, Dijkman PM, Lea WA, van den Bogaart G, Jerabek-Willemsen M, Lazic A, et al Microscale thermophoresis quantifies biomolecular interactions under previously challenging conditions. Methods. 2013;59(3):301–15. 10.1016/j.ymeth.2012.12.005 . 23270813 PMC3644557

[59] Qian C, Li S, Jakoncic J, Zeng L, Walsh MJ, Zhou MM. Structure and hemimethylated CpG binding of the SRA domain from human UHRF1. J Biol Chem. 2008;283(50):34490–4. 10.1074/jbc.C800169200 . 18945682 PMC2596396

[60] Greiner VJ, Kovalenko L, Humbert N, Richert L, Birck C, Ruff M, et al Site-Selective Monitoring of the Interaction of the SRA Domain of UHRF1 with Target DNA Sequences Labeled with 2-Aminopurine. Biochemistry. 2015;54(39):6012–20. 10.1021/acs.biochem.5b00419 . 26368281

[61] Kilin V, Gavvala K, Barthes NPF, Michel BY, Shin D, Boudier C, et al Dynamics of Methylated Cytosine Flipping by UHRF1. Journal of the American Chemical Society. 2017;139(6):2520–8. 10.1021/jacs.7b00154 28112929 PMC5335914

[62] Helabad MB, Kanaan N, Imhof P. Base Flip in DNA Studied by Molecular Dynamics Simulations of Differently-Oxidized Forms of Methyl-Cytosine. International Journal of Molecular Sciences. 2014;15(7):11799–816. 10.3390/ijms150711799 24995694 PMC4139815

[63] Szulik MW, Pallan PS, Nocek B, Voehler M, Banerjee S, Brooks S, et al Differential Stabilities and Sequence-Dependent Base Pair Opening Dynamics of Watson–Crick Base Pairs with 5-Hydroxymethylcytosine, 5-Formylcytosine, or 5-Carboxylcytosine. Biochemistry. 2015;54(5):1294–305. 10.1021/bi501534x 25632825 PMC4325598

[64] Huang N, Banavali NK, MacKerell AD. Protein-facilitated base flipping in DNA by cytosine-5-methyltransferase. Proceedings of the National Academy of Sciences. 2003;100(1):68. 10.1073/pnas.0135427100 PMC140885 12506195

[65] Rajakumara E, Law JA, Simanshu DK, Voigt P, Johnson LM, Reinberg D, et al A dual flip-out mechanism for 5mC recognition by the Arabidopsis SUVH5 SRA domain and its impact on DNA methylation and H3K9 dimethylation in vivo. Genes Dev. 2011;25(2):137–52. 10.1101/gad.1980311 . 21245167 PMC3022260

[66] Arita K, Isogai S, Oda T, Unoki M, Sugita K, Sekiyama N, et al Recognition of modification status on a histone H3 tail by linked histone reader modules of the epigenetic regulator UHRF1. Proc Natl Acad Sci U S A. 2012;109(32):12950–5. 10.1073/pnas.1203701109 . 22837395 PMC3420164

[67] Bianchi C, Zangi R. Dual base-flipping of cytosines in a CpG dinucleotide sequence. Biophysical Chemistry. 2014;187–188:14–22. 10.1016/j.bpc.2013.12.005 . 24469333

[68] Hashimoto H, Horton JR, Zhang X, Cheng X. UHRF1, a modular multi-domain protein, regulates replication-coupled crosstalk between DNA methylation and histone modifications. Epigenetics. 2009;4:8–14. 10.4161/epi.4.1.7370 . 19077538 PMC2661099

[69] Bronner C, Fuhrmann G, Chédin FL, Macaluso M, Dhe-Paganon S. UHRF1 Links the Histone code and DNA Methylation to ensure Faithful Epigenetic Memory Inheritance. Genetics & epigenetics. 2010;2009(2):29–36. 21643543 PMC3106981

[70] Grin I, Ishchenko AA. An interplay of the base excision repair and mismatch repair pathways in active DNA demethylation. Nucleic Acids Res. 2016;44(8):3713–27. 10.1093/nar/gkw059 . 26843430 PMC4856981

[71] Kohli RM, Zhang Y. TET enzymes, TDG and the dynamics of DNA demethylation. Nature. 2013;502:472–9. 10.1038/nature12750 . 24153300 PMC4046508

[72] Bochtler M, Kolano A, Xu GL. DNA demethylation pathways: Additional players and regulators. Bioessays. 2017;39(1):1–13. 10.1002/bies.201600178 . 27859411

[73] Maiti A, Drohat AC. Thymine DNA glycosylase can rapidly excise 5-formylcytosine and 5-carboxylcytosine: potential implications for active demethylation of CpG sites. J Biol Chem. 2011;286(41):35334–8. 10.1074/jbc.C111.284620 . 21862836 PMC3195571

[74] Muller U, Bauer C, Siegl M, Rottach A, Leonhardt H. TET-mediated oxidation of methylcytosine causes TDG or NEIL glycosylase dependent gene reactivation. Nucleic Acids Res. 2014;42(13):8592–604. 10.1093/nar/gku552 . 24948610 PMC4117777

[75] Weber AR, Krawczyk C, Robertson AB, Kusnierczyk A, Vagbo CB, Schuermann D, et al Biochemical reconstitution of TET1-TDG-BER-dependent active DNA demethylation reveals a highly coordinated mechanism. Nat Commun. 2016;7:10806 10.1038/ncomms10806 . 26932196 PMC4778062

[76] Luo T, Cui S, Bian C, Yu X. Uhrf2 is important for DNA damage response in vascular smooth muscle cells. Biochem Biophys Res Commun. 2013;441(1):65–70. 10.1016/j.bbrc.2013.10.018 . 24134842 PMC3985570

[77] Mistry H, Tamblyn L, Butt H, Sisgoreo D, Gracias A, Larin M, et al UHRF1 is a genome caretaker that facilitates the DNA damage response to gamma-irradiation. Genome Integr. 2010;1(1):7 10.1186/2041-9414-1-7 . 20678257 PMC2914011

[78] Tian Y, Paramasivam M, Ghosal G, Chen D, Shen X, Huang Y, et al UHRF1 contributes to DNA damage repair as a lesion recognition factor and nuclease scaffold. Cell Reports. 2015;10:1957–66. 10.1016/j.celrep.2015.03.038 . 25818288 PMC4748712

[79] Laget S, Miotto B, Chin HG, Esteve PO, Roberts RJ, Pradhan S, et al MBD4 cooperates with DNMT1 to mediate methyl-DNA repression and protects mammalian cells from oxidative stress. Epigenetics. 2014;9(4):546–56. 10.4161/epi.27695 . 24434851 PMC4121365

[80] O'Hagan HM, Wang W, Sen S, Destefano Shields C, Lee SS, Zhang YW, et al Oxidative damage targets complexes containing DNA methyltransferases, SIRT1, and polycomb members to promoter CpG Islands. Cancer Cell. 2011;20(5):606–19. 10.1016/j.ccr.2011.09.012 . 22094255 PMC3220885

[81] Kamiya H, Tsuchiya H, Karino N, Ueno Y, Matsuda A, Harashima H. Mutagenicity of 5-Formylcytosine, an Oxidation Product of 5-Methylcytosine, in DNA in Mammalian Cells1. The Journal of Biochemistry. 2002;132(4):551–5. 10.1093/oxfordjournals.jbchem.a003256 12359069

[82] Shibutani T, Ito S, Toda M, Kanao R, Collins LB, Shibata M, et al Guanine- 5-carboxylcytosine base pairs mimic mismatches during DNA replication. Sci Rep. 2014;4:5220 10.1038/srep05220 . 24910358 PMC4048885

[83] Kim KY, Tanaka Y, Su J, Cakir B, Xiang Y, Patterson B, et al Uhrf1 regulates active transcriptional marks at bivalent domains in pluripotent stem cells through Setd1a. Nat Commun. 2018;9(1):2583 10.1038/s41467-018-04818-0 . 29968706 PMC6030064

[84] Verma N, Pan H, Dore LC, Shukla A, Li QV, Pelham-Webb B, et al TET proteins safeguard bivalent promoters from de novo methylation in human embryonic stem cells. Nat Genet. 2018;50(1):83–95. 10.1038/s41588-017-0002-y . 29203910 PMC5742051

[85] Deplus R, Delatte B, Schwinn MK, Defrance M, Mendez J, Murphy N, et al TET2 and TET3 regulate GlcNAcylation and H3K4 methylation through OGT and SET1/COMPASS. EMBO J. 2013;32(5):645–55. 10.1038/emboj.2012.357 . 23353889 PMC3590984

