# Supplementary material for: Systematic analysis of the binding behaviour of UHRF1 towards different methyl- and carboxylcytosine modification patterns at CpG dyads
Source: PLoS One. 2020 Feb 21;15(2):e0229144. doi: 10.1371/journal.pone.0229144 (PMC7034832; doi:10.1371/journal.pone.0229144)

Information about raw images:

Page 1 shows an overview of all performed EMSA experiments for UHRF1 and UHRF2. Quantitation has been performed as described in the Materials and Methods section. Boxplots in Fig 2c and S3 Fig are based on these numbers.

Pages 2 to 10 show minimally cropped original scans of all gels used for the study; neither brightness nor contrast has been altered. Gels have been marked with empty lanes to avoid confusion during handling. These empty lanes have been ignored during analysis and have been removed in the main figure (Fig 2b).

Each scan has 3 channels (shown individually):

- the 488-channel represents the GFP-coupled protein
- the 550-channel depicts the signal of the modified DNA oligonucleotide (modifications are indicated in the figure)
- the 647-channel shows the signal of the unmodified control DNA

UHRF1	C/C	hemi-mC/C	hemi-hmC/C	hemi-fC/C	hemi-caC/C	sym. mC/C	sym. hmC/C	sym. fC/C	sym. caC/C
**Exp1**	0.85					1.06	1.34		1.71
**Exp2**	1.20					1.07	1.39	1.19	1.71
**Exp3**									1.81
**Exp4**	0.91					1.51	1.18		1.78
**Exp5**	0.99					1.41	0.75	0.95	1.88
**Exp6**	0.63					1.20	0.81	0.81	1.71
**Exp7**	1.23	1.63		1.12	1.12				
**Exp8**	0.82	0.94	0.92	0.84	1.09				
**Exp9**	1.29	2.04	1.65	1.75	1.51				
**Exp10**	1.22	1.38	0.56	0.70	1.19				
**mean**	1.02	1.50	1.04	1.10	1.23	1.25	1.09	0.98	1.77
**stddev**	0.23	0.46	0.55	0.47	0.19	0.20	0.30	0.19	0.07
**norm.**	1.00	1.47	1.02	1.08	1.21	1.23	1.08	0.97	1.74
**stddev**	0.32	0.56	0.59	0.52	0.33	0.34	0.38	0.28	0.39
**p-value**	1.00E+00	2.45E-02	9.09E-01	6.64E-01	1.38E-01	8.23E-02	5.94E-01	8.20E-01	3.17E-06
**#Rep**	9	4	3	4	4	5	5	3	6

UHRF2	C/C	hemi-mC/C	hemi-hmC/C	hemi-fC/C	hemi-caC/C	sym. mC/C	sym. hmC/C	sym. fC/C	sym. caC/C
**Exp1**	1.51					0.99	1.78	2.03	2.48
**Exp2**	1.42					1.17	1.43	1.42	2.23
**Exp3**	1.32					1.22	1.35	1.32	2.35
**Exp4**	1.29	1.25	1.32	0.94					
**Exp5**	0.87	0.78	0.78	0.77	0.90				
**Exp6**	0.71	0.86		0.77	0.87				
**Exp7**	1.17	0.90	0.88	1.12	1.01				
**mean**	1.18	0.94	0.99	0.90	0.92	1.13	1.52	1.59	2.35
**stddev**	0.29	0.21	0.29	0.17	0.08	0.12	0.23	0.38	0.12
**norm.**	1.00	0.80	0.84	0.76	0.78	0.95	1.28	1.34	1.99
**stddev**	0.35	0.26	0.32	0.23	0.20	0.26	0.37	0.46	0.50
**p-value**	1.00E+00	1.86E-01	3.72E-01	1.11E-01	1.79E-01	7.64E-01	1.17E-01	1.02E-01	1.89E-04
**#Rep**	7	4	3	4	3	3	3	3	3

Legend

protein serial dilution

excluded due to lack of relevance

excluded due to quality concerns or technical reasons

gel belongs to different experiment and is labeled there

UHRF1 symmetric modifications, Experiments 1 + 2

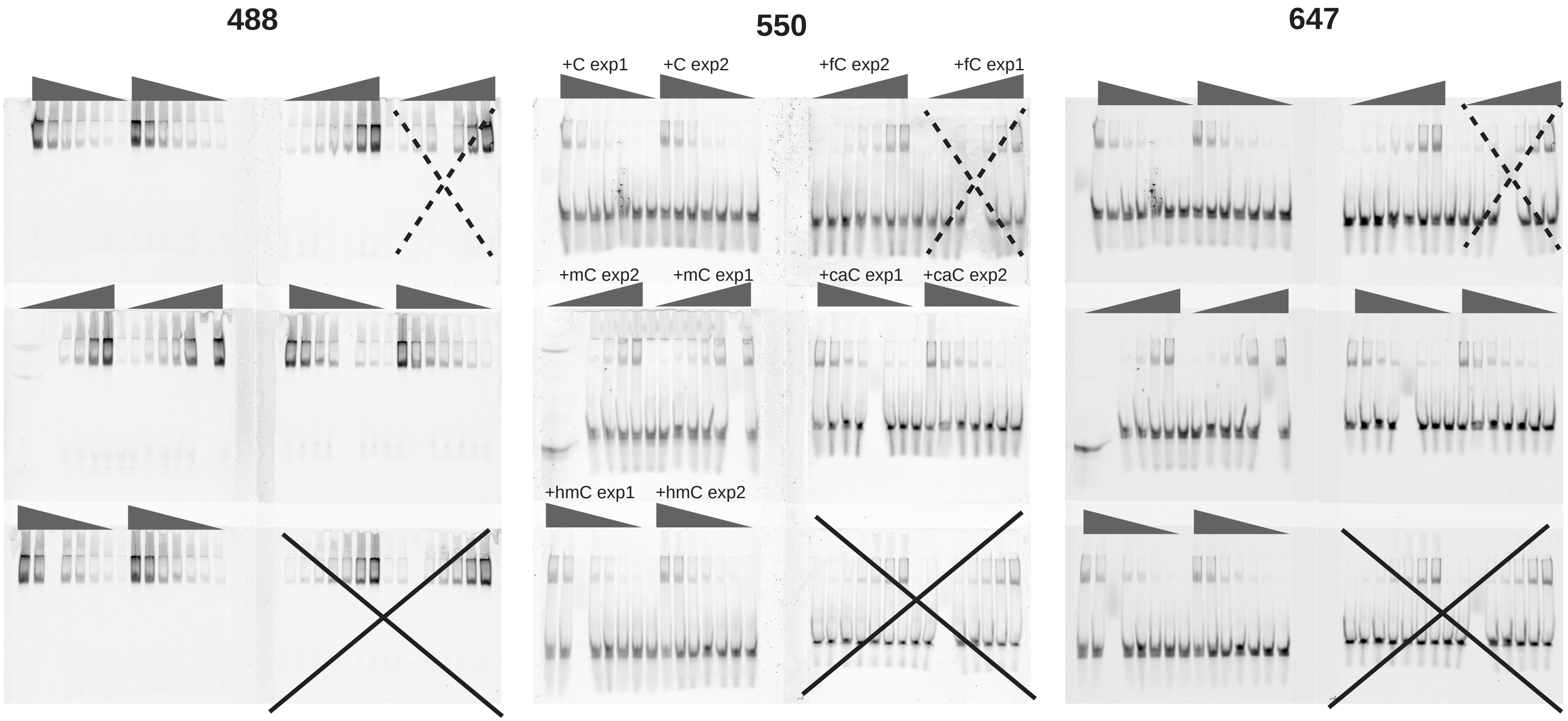

UHRF1 symmetric modifications, Experiments 3 + 6

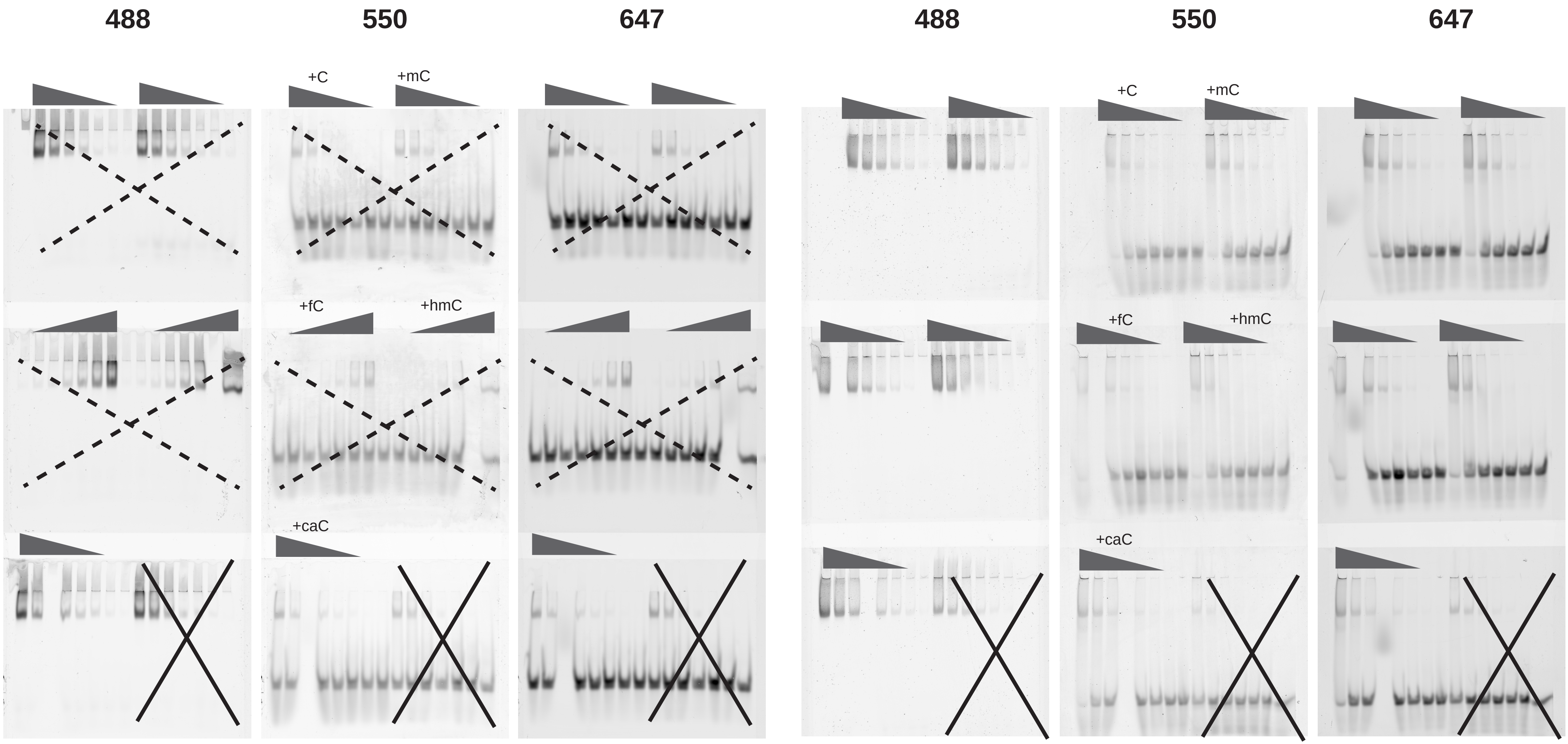

UHRF1 symmetric modifications, Experiments 4 + 5

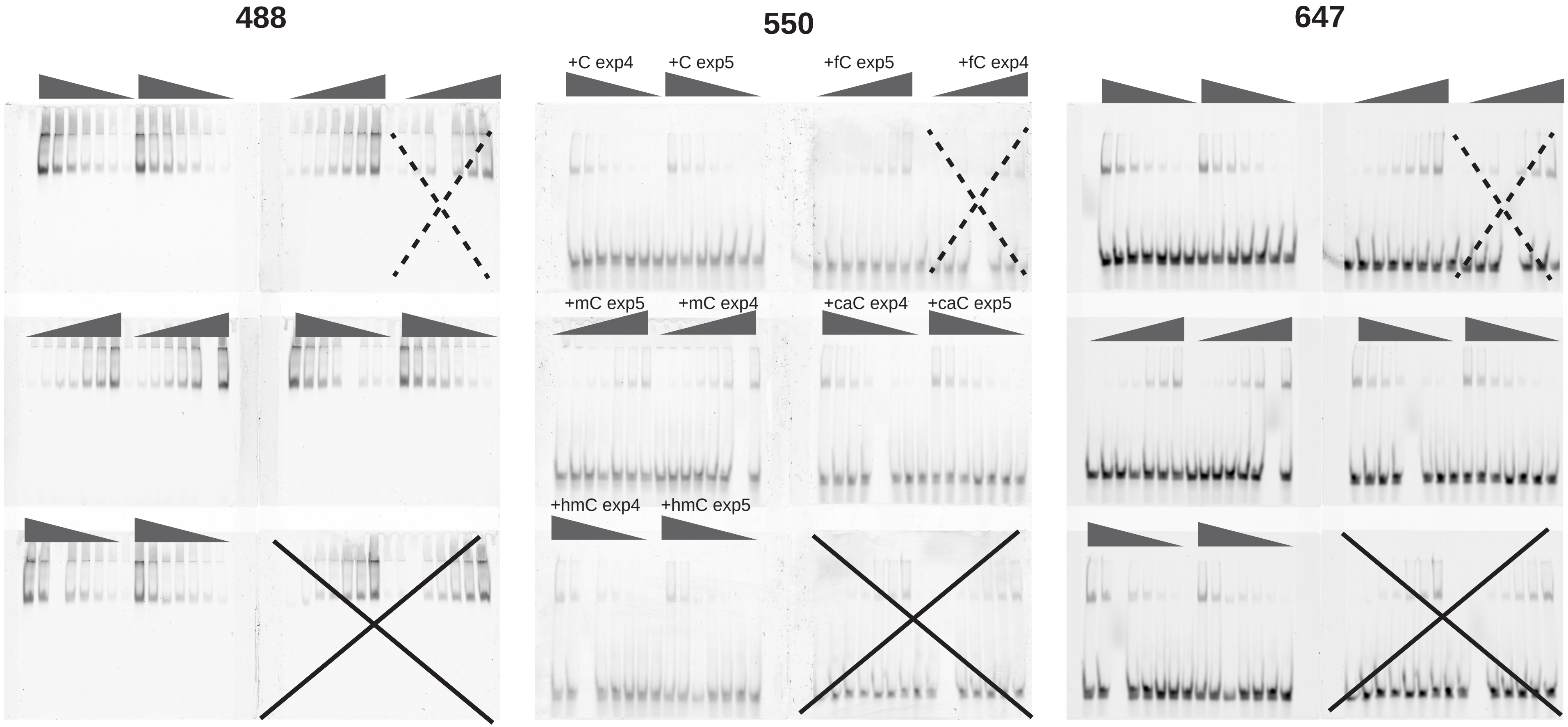

UHRF1 hemi-modifications, Experiments 7 + 8

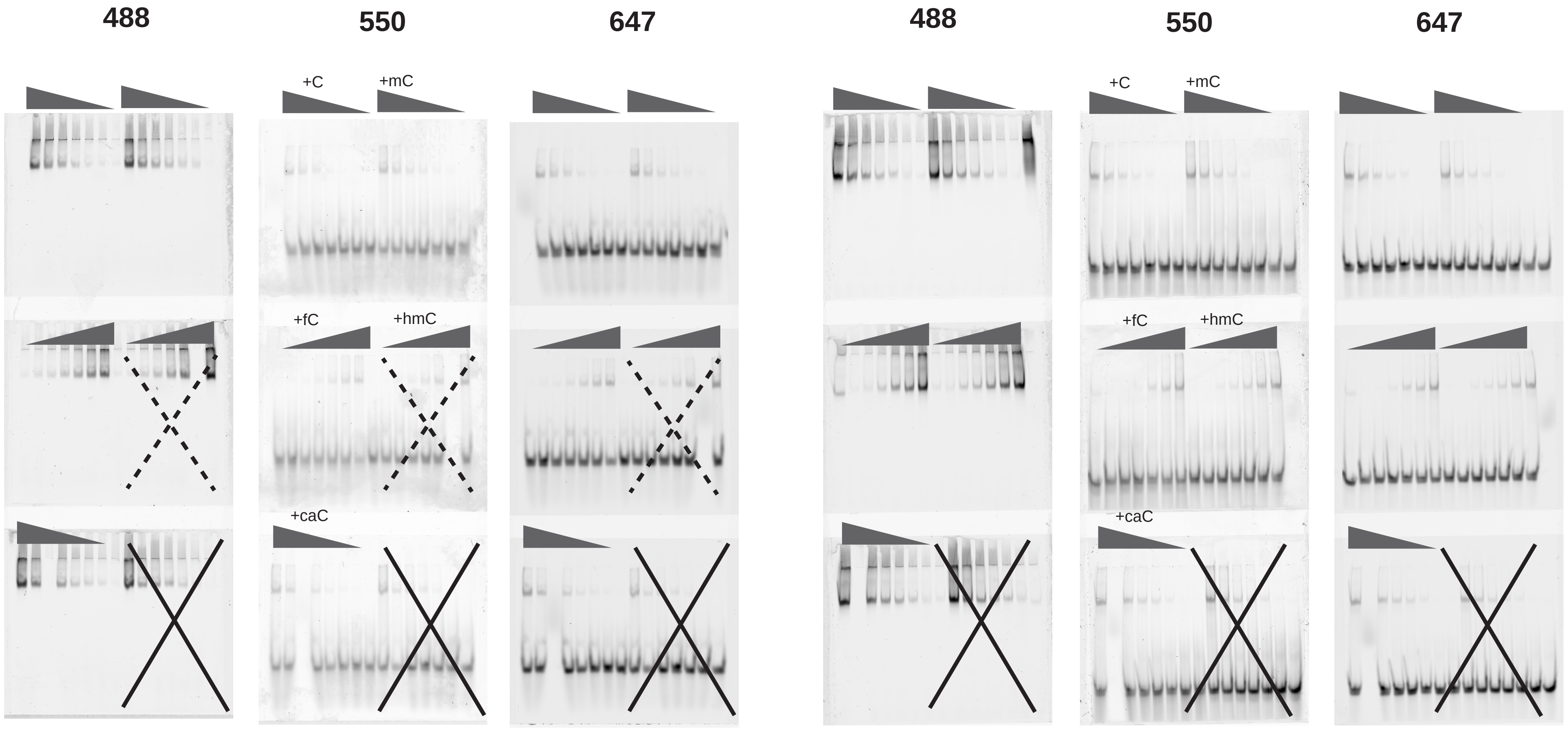

UHRF1 hemi-modifications, Experiments 9 + 10

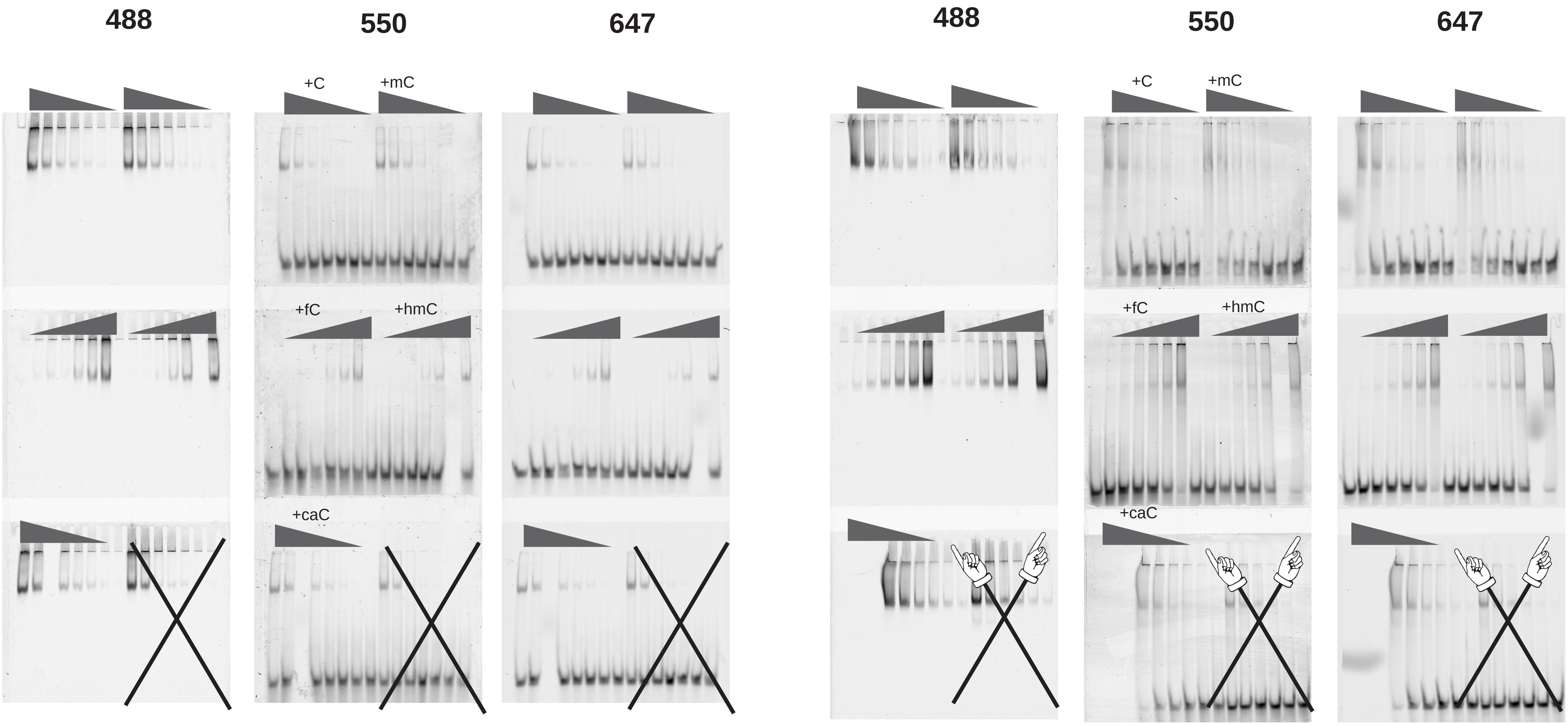

UHRF2 symmetric modifications, Experiment 1

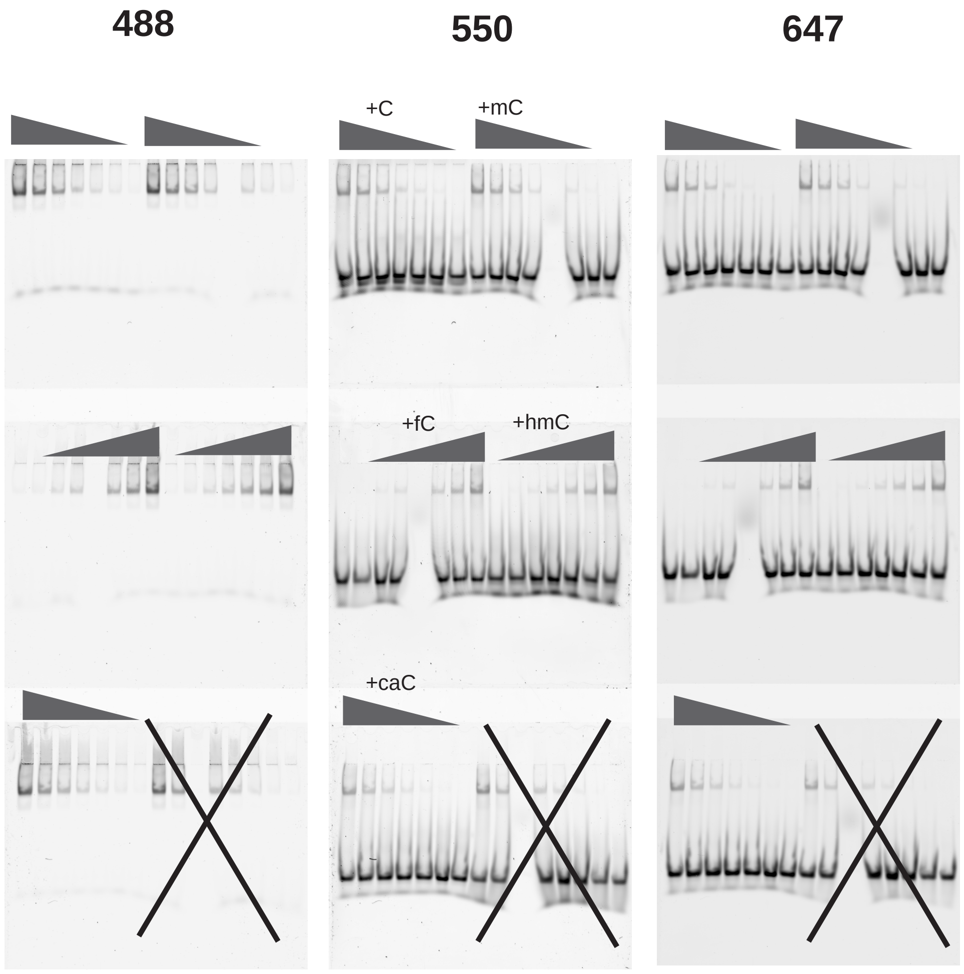

UHRF2 symmetric modifications, Experiments 2 + 3

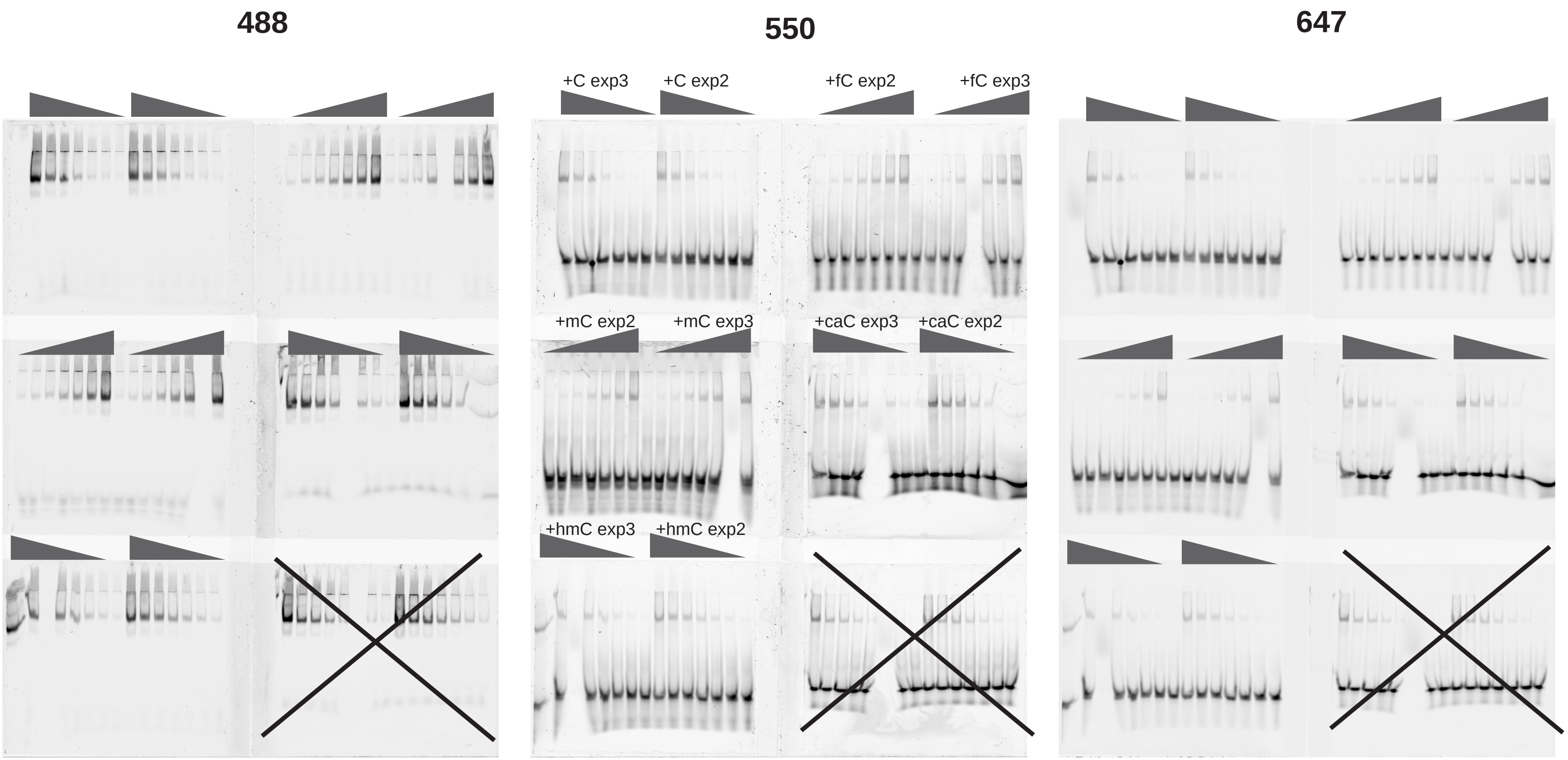

UHRF2 hemi-modifications, Experiments 4 + 5

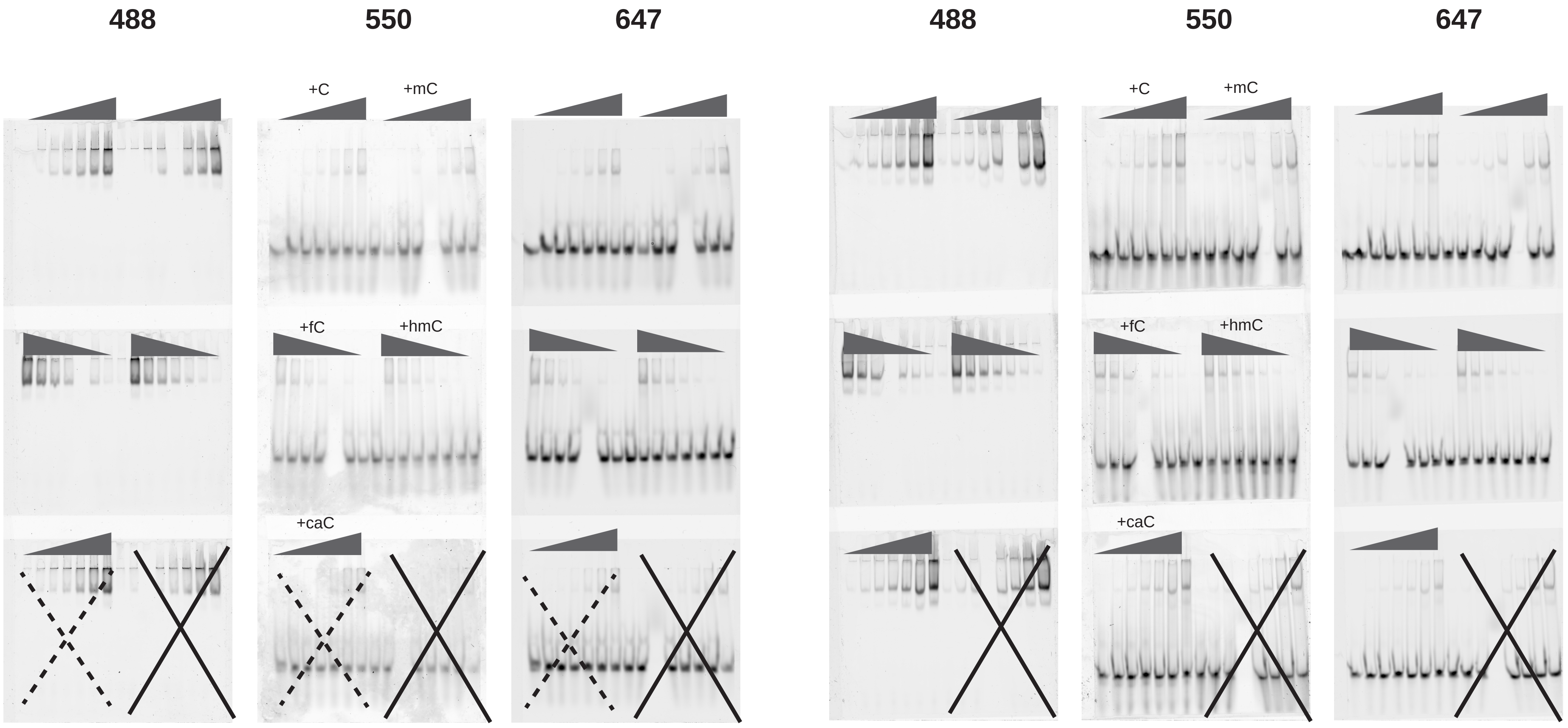

UHRF2 hemi-modifications, Experiments 6 + 7

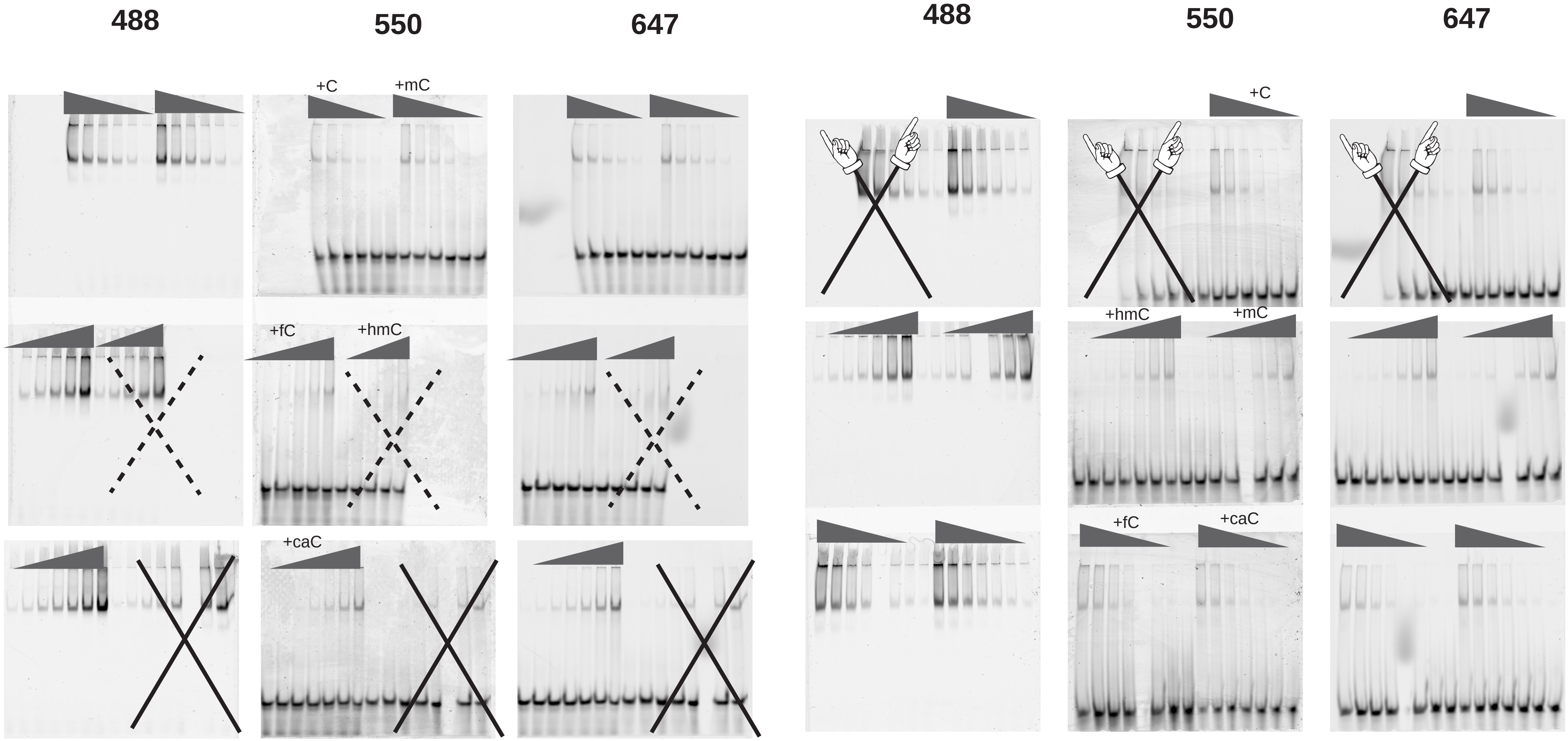

Supplement: S1 Fig — All raw gel scans that have been used to generate the EMSA results presented in Fig 2b/2c and S5 Fig. An overview of all individual quantitative values and the corresponding statistics is provided on page 1. (PDF) [file pone.0229144.s001.pdf]
