# Supplementary figures and images for: Systematic analysis of the binding behaviour of UHRF1 towards different methyl- and carboxylcytosine modification patterns at CpG dyads
Source: PLoS One. 2020 Feb 21;15(2):e0229144. doi: 10.1371/journal.pone.0229144 (PMC7034832; doi:10.1371/journal.pone.0229144)

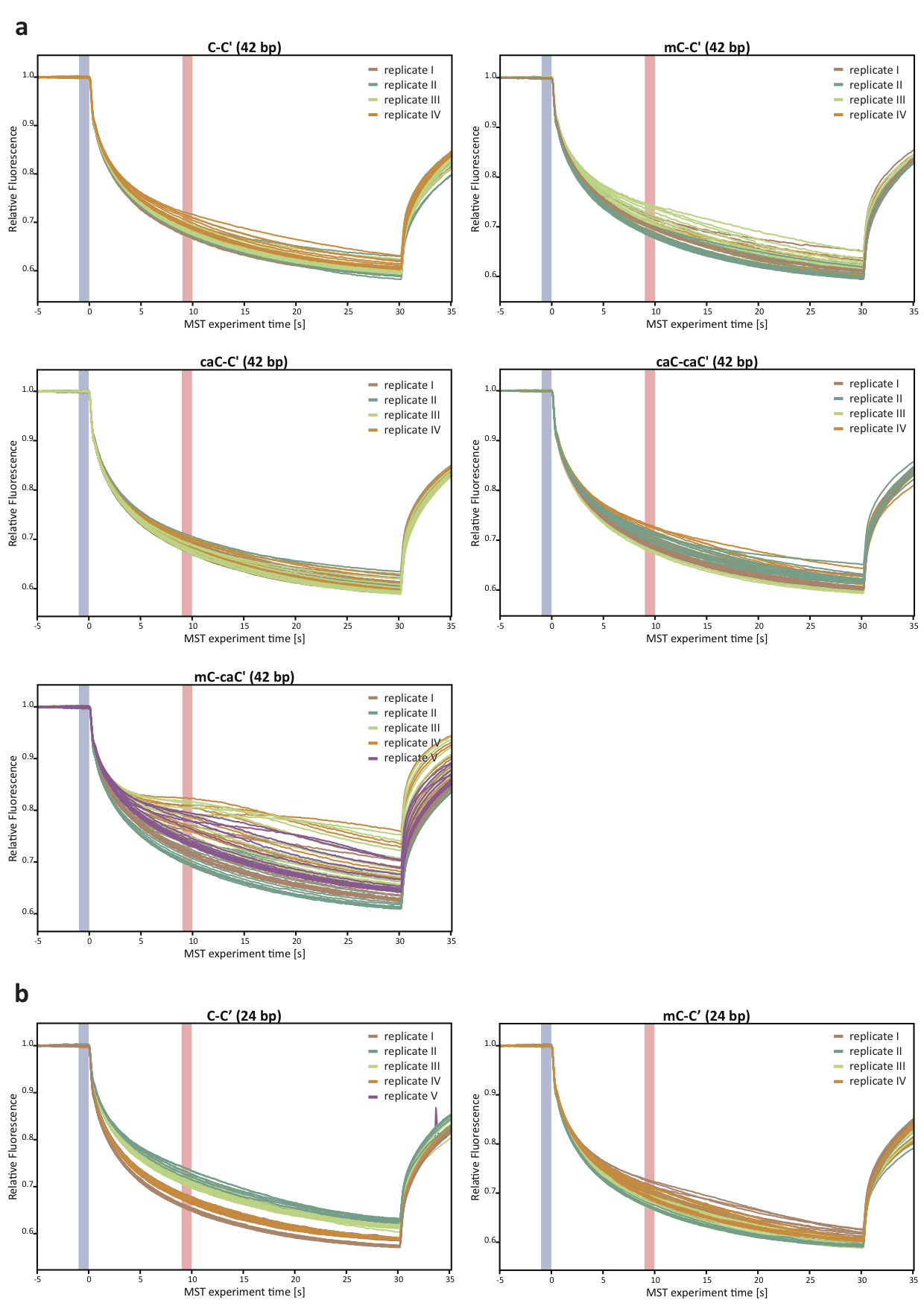

Supplement: S2 Fig — Fluorescence traces that have been used to generate the binding curves in Fig 3. Traces are shown individually for all modifications and are coloured by experimental replicate. Blue and red bars indicate the time points that were used for the analysis; blue: tcold (pre infra-red laser), red: thot (post infra-red laser). (TIF) [file pone.0229144.s002.tif]

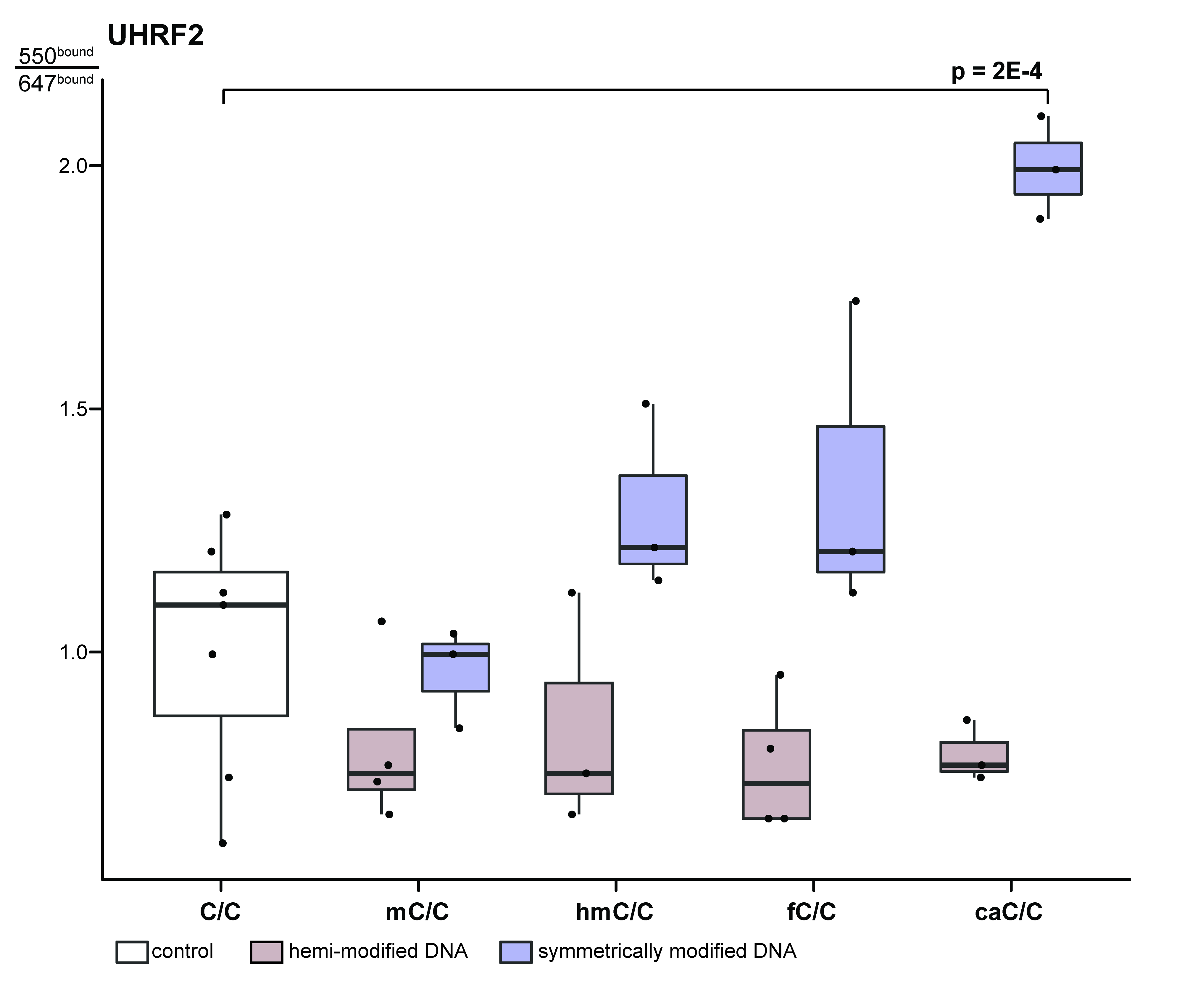

Supplement: S5 Fig — Quantitation of the bound fraction of EMSAs of wild type UHRF2-GFP with 42 bp DNA oligonucleotides carrying different cytosine modifications. Experiments and analyses have been performed as in Fig 2. (TIF) [file pone.0229144.s005.tif]

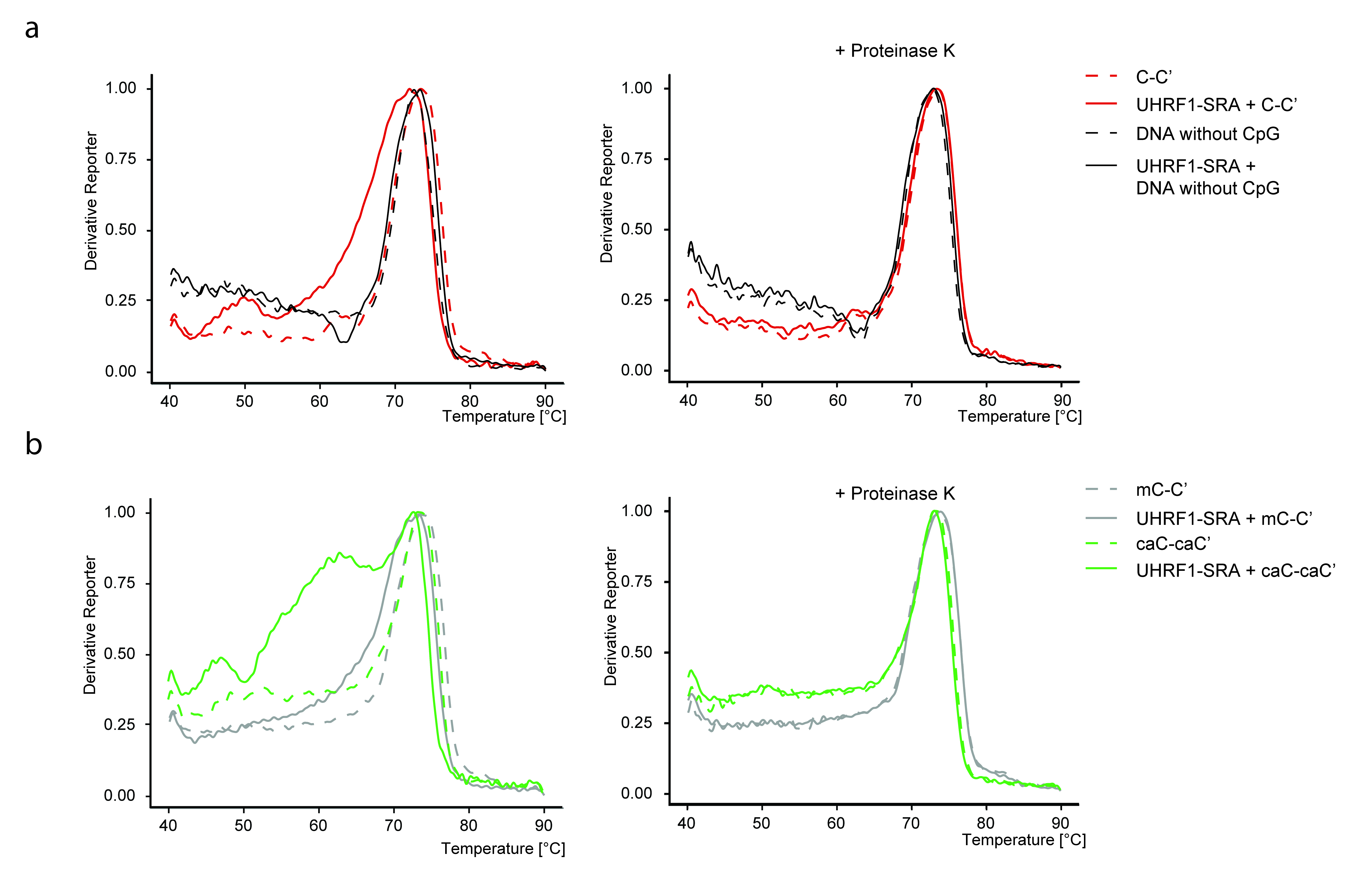

Supplement: S6 Fig — (a) The melting temperature of double-stranded DNA containing C-C’ in a CpG context (red) or no CpG site (black) with (solid lines) or without (dotted lines) a 5-fold excess of the SRA domain of UHRF1, measured using high resolution melting temperature (HRM) analysis. As control, proteins were digested by proteinase K before HRM analysis (right panel). Experiments were performed independently three times; one representative experiment is depicted as average of three technical replicates. (b) Melting temperatures as in (a) with DNA harbouring symmetric caC (green) or hemi-mC (gray) at the central CpG site. (TIF) [file pone.0229144.s006.tif]

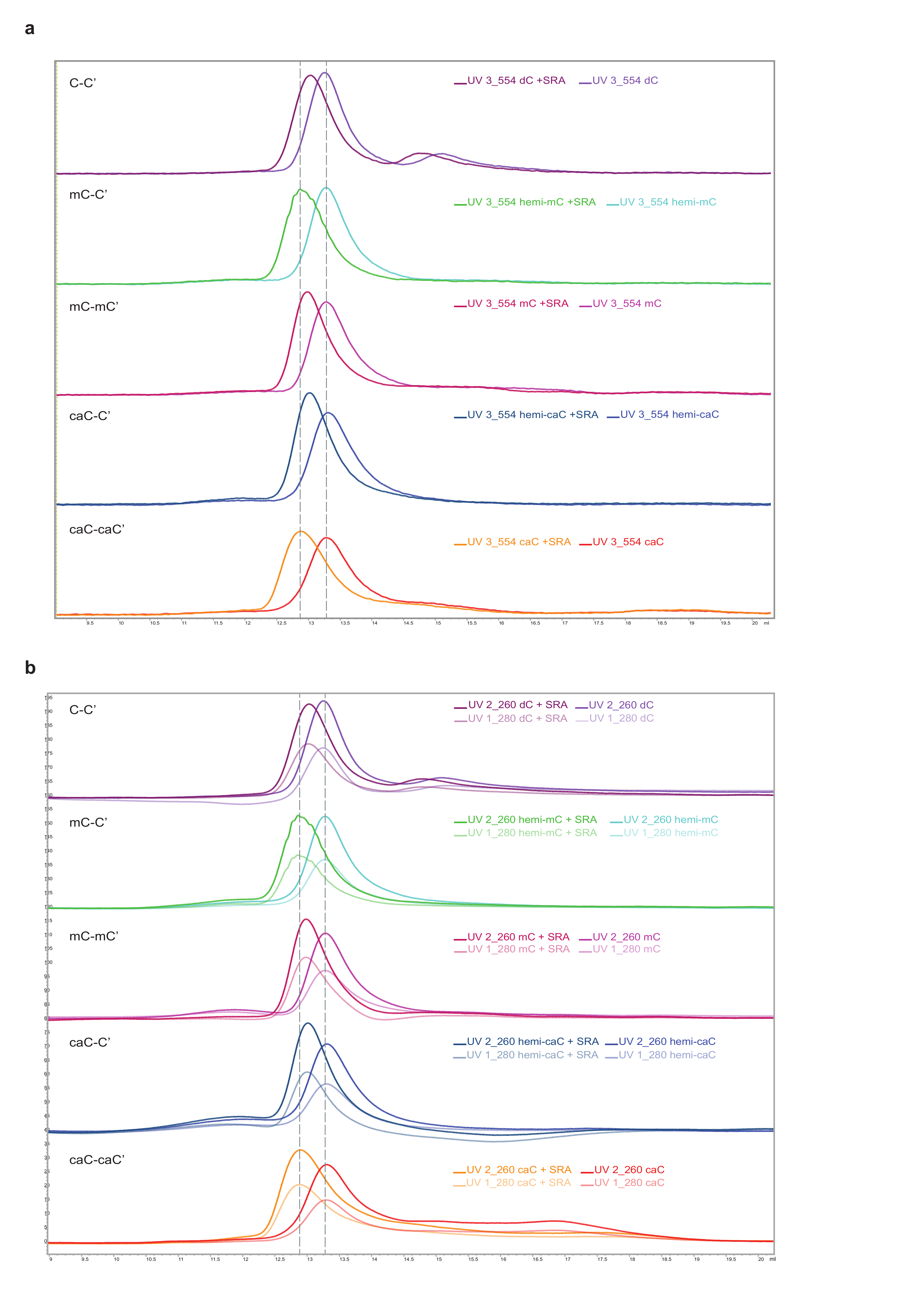

Supplement: S7 Fig — To test for different binding stoichiometries of the SRA domain towards differentially modified DNA, ATTO550-labeled DNA oligonucleotides were incubated with a 10-fold excess of SRA. Size exclusion chromatograms of analyzed DNA oligonucleotides at an absorbance of 554 nm (a) and 260nm/280nm (b) show a clear and comparable shift in retention time for the SRA-bound DNA (left peaks) compared to free DNA (right peaks). (TIF) [file pone.0229144.s007.tif]

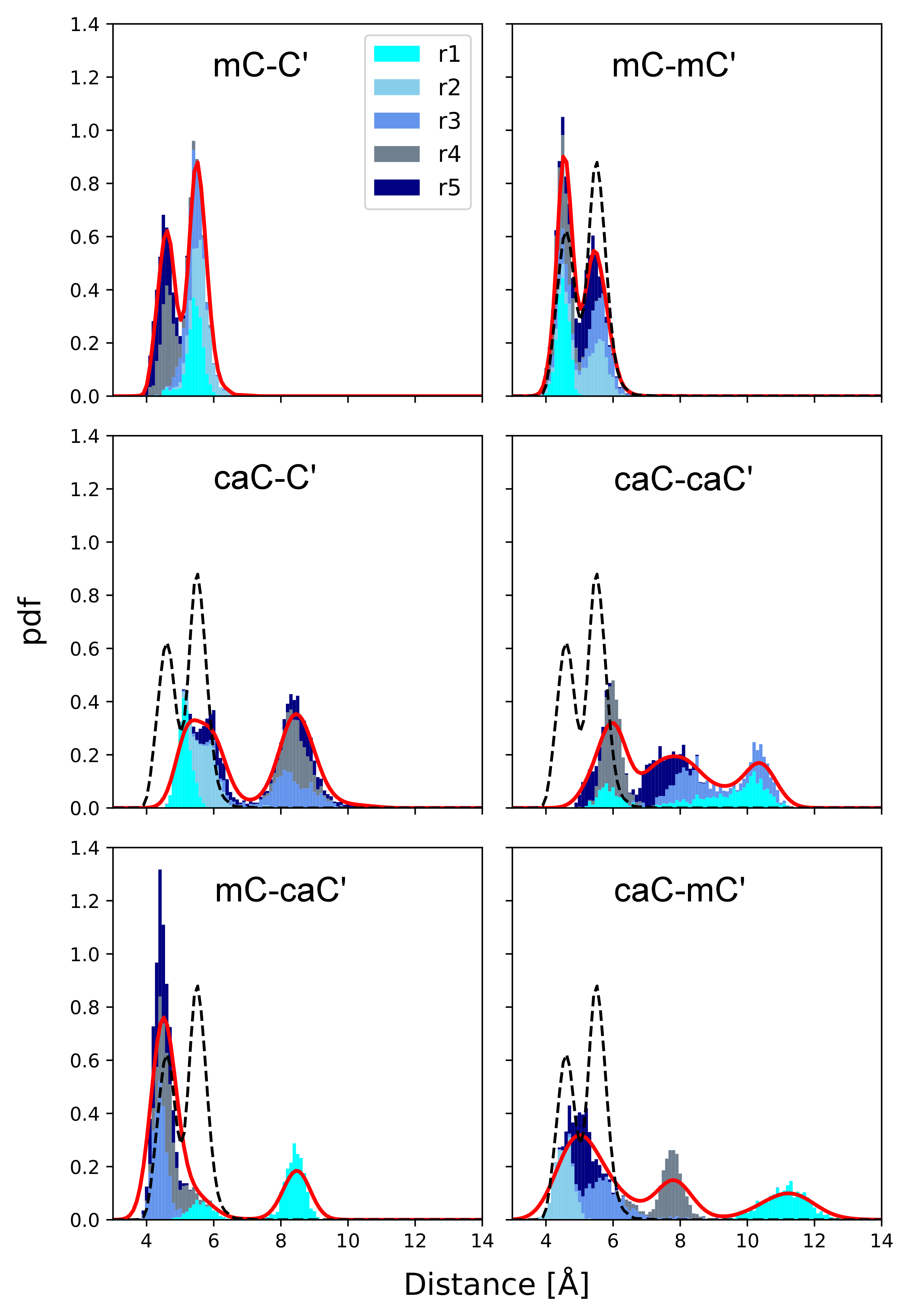

Supplement: S8 Fig — Individual replicas are shown as separate bars stacked on top of each other. Distances were measured between the geometric centres of the phenyl and pyrimidine rings. Red lines show a gaussian kernel estimate of the probability density function (pdf). The estimated pdf of the mC-C’ system is shown as black dashed lines. (TIF) [file pone.0229144.s008.tif]

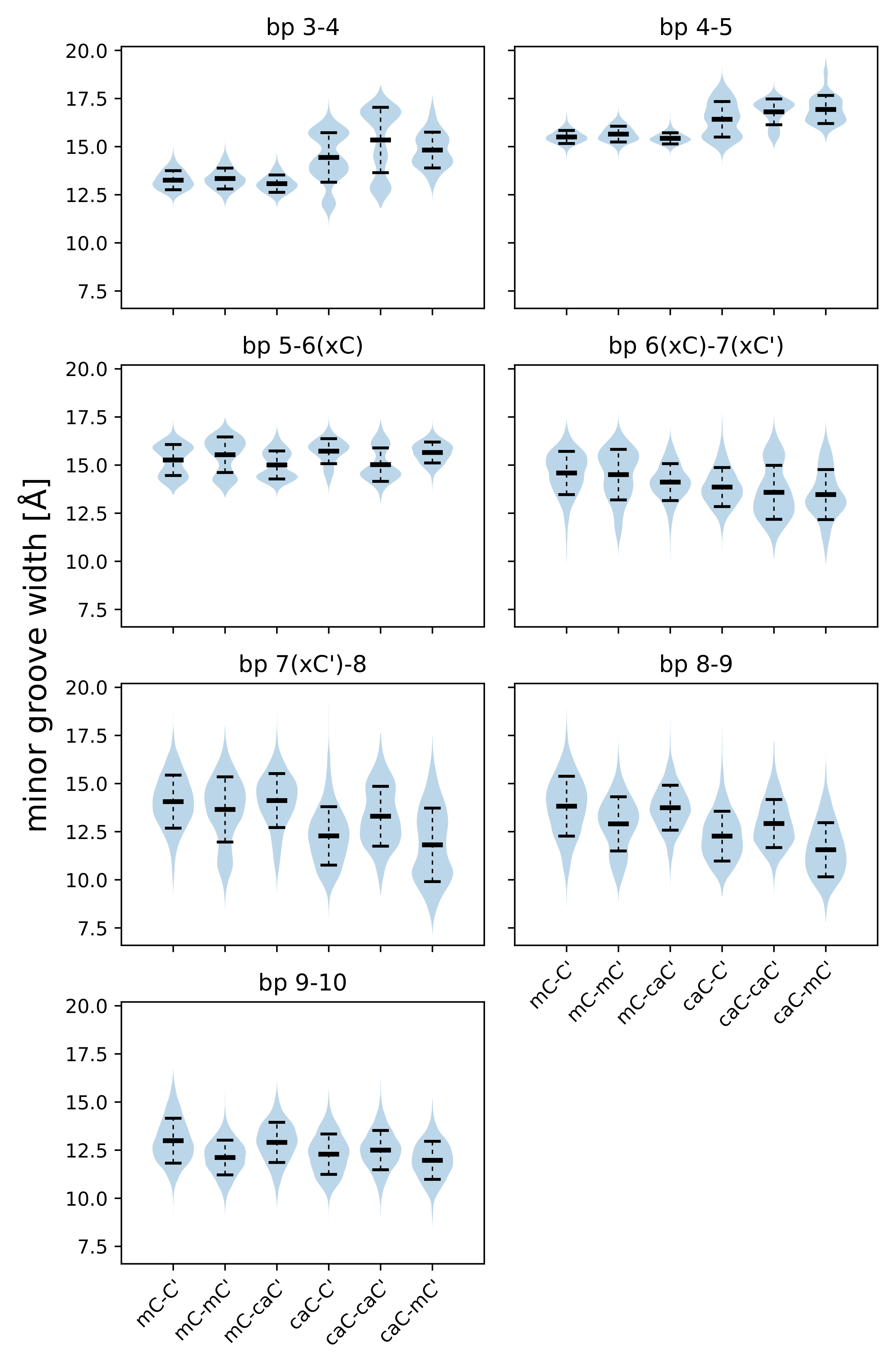

Supplement: S9 Fig — Blue faces represent gaussian kernel estimates of the underlying values. Black bars show distribution means and standard deviations. (TIF) [file pone.0229144.s009.tif]

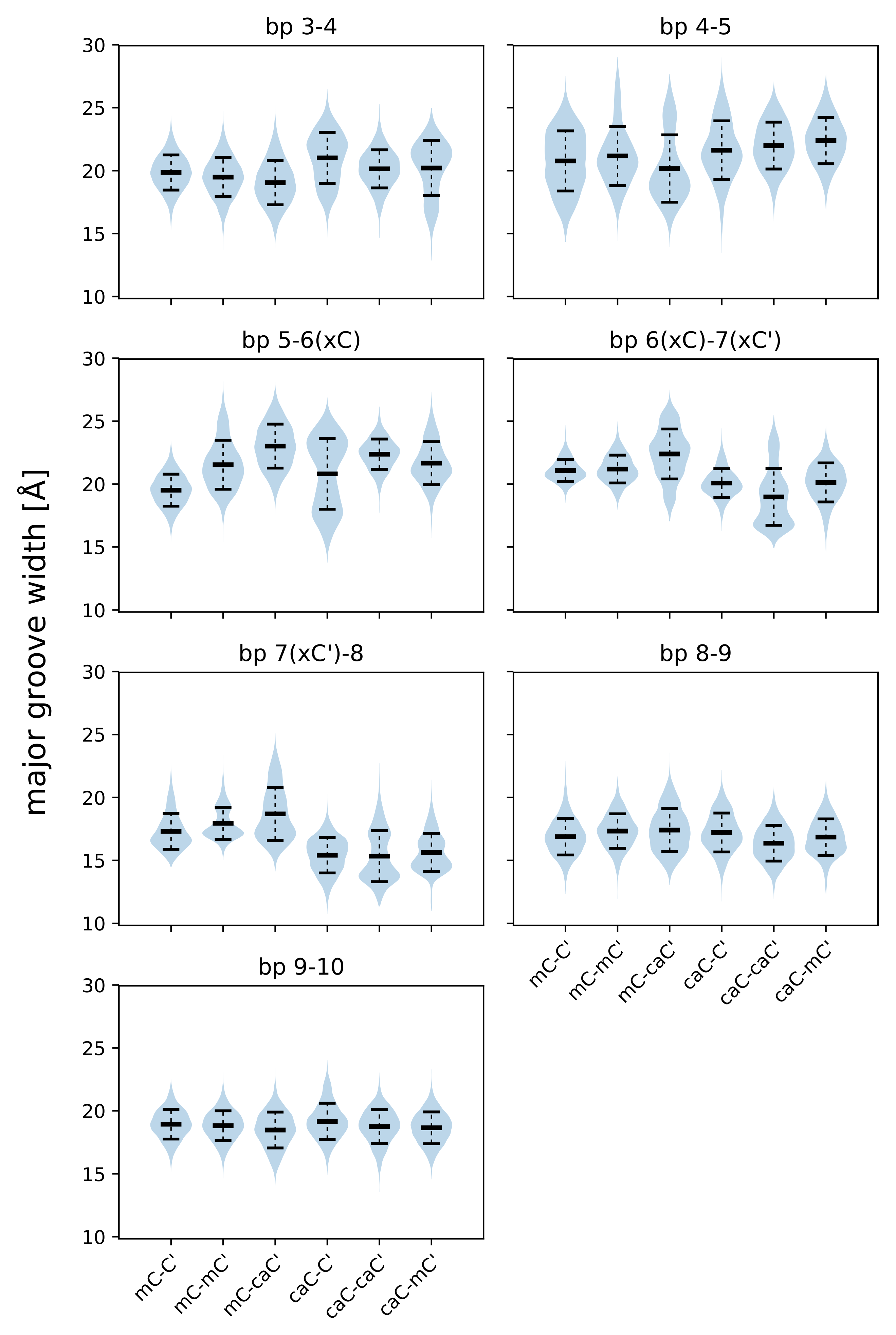

Supplement: S10 Fig — Blue faces represent gaussian kernel estimates of the underlying values. Black bars show distribution means and standard deviations. (TIF) [file pone.0229144.s010.tif]
