# Supplementary material for: Systematic analysis of the binding behaviour of UHRF1 towards different methyl- and carboxylcytosine modification patterns at CpG dyads
Source: PLoS One. 2020 Feb 21;15(2):e0229144. doi: 10.1371/journal.pone.0229144 (PMC7034832; doi:10.1371/journal.pone.0229144)
Supplement: S1 Text — (DOCX) [file pone.0229144.s011.docx]

**S1 Text.** **Additional Experimental Procedures.**

**Protein purification**

10 p150 dishes of HEK293T cells were transfected with the expression construct, grown for 2 - 3 days in DMEM including 10 % FCS, and harvested by physical detachment. Cells were lysed for 30 min on ice in lysis buffer (50 mM NaH_2_PO_4_, pH = 7.5, 150 mM NaCl, 10 mM imidazole, 0.5 % Tween-20, 0.5 mM EDTA, 1 g/l DNase I, 2 mM MgCl_2_, 0.5 mM CaCl_2_, 1 mM PMSF, 1x mammalian protease inhibitor (SERVA)). Cleared lysates were incubated with NiNTA-coupled GFP-binder [1] for 1 - 2 hours at 4°C. After washing, proteins were eluted with 250 mM imidazole (in 50 mM NaH_2_PO_4_, pH = 7.5, 300 mM NaCl, 0.05 % Tween-20). Buffers were exchanged to binding buffer (20 mM TrisHCl, pH = 7.5, 150 mM NaCl, 0.5 mM EDTA, 1 mM DTT) via PD-10 columns (GE Healthcare Life Sciences). Protein quantification was performed based on GFP intensities with an Infinite M1000 plate reader (Tecan).

For purification of the SRA domain from E. coli, expression cultures were grown at 37 °C and protein expression was induced with 0.5 mM IPTG when OD_600_ reached 0.6-0.8. After 3 hours, cells were lysed in binding buffer B (30 mM Tris-HCl pH 7.5, 300 mM NaCl, 10 mM imidazole, 5 mM β-mercaptoethanol) supplemented with 1 mM PMSF, 100 µg/ml lysozyme and 25 µg/ml DNase under constant rotation. The lysate was sonicated and centrifuged at 20,000 g for 20 min. Inclusion bodies were cleared from cell debris by resuspending pelleted matter in wash buffer W (Buffer B + 0.5 % Triton X 100) and subsequent centrifugation at 20,000 g for 20 min. All purification steps were carried out on an Äkta Purifier system (GE Healthcare). Highly pure inclusion bodies were dissolved in denaturation buffer D (Buffer B + 8 M Urea) and loaded onto a HisTrap FF crude 1 mL column (GE Healthcare). Immobilized protein was refolded on column by applying a linear gradient from 100 % buffer D to 100 % Buffer B over 20 column volumes. Refolded soluble protein was eluted from the column with a linear gradient from 100 % buffer B to 100 % elution buffer E (30 mM Tris-HCl pH 7.5, 300 mM NaCl, 500 mM imidazole, 5 mM β-mercaptoethanol) over five column volumes. Peak fractions were collected and analysed by SDS-PAGE for purity and protein integrity.

**Melting Temperature Analysis**

To test the effect of SRA proteins on the stability of double-stranded DNA, we incubated 2.5 µM of the purified SRA domain, 0.5 µM of the 42 bp double-stranded DNA oligonucleotide and 1x reaction buffer (50 mM Tris-HCl, 0.5 mM β-ME, 10 mM EDTA, pH 7.5) in a total volume of 10 µL at 37°C for 30 minutes. Then, 10 µL of SYBR Green I Mix (Platinum SYBR Green qPCR Super Mix, 1:4 dilution, Invitrogen) was added to each reaction and high-resolution melting temperature analysis was performed in the StepOnePlusTM Real-Time PCR system (Applied Biosystems) by increasing temperature from 40°C to 90°C with 0.1°C steps. As a control, SRA proteins were digested with 20 µg proteinase K (Sigma-Aldrich) at 50°C for 1 hour before adding SYBR Green I Mix. To visualize the melting temperature of DNA, the derivative values (SYBR Green I fluorescence against the temperature) were exported from StepOne software 2.1 (Applied Biosystems) and further plotted using RStudio (0.98.1087).

**Analytic size exclusion chromatography**

To test for different binding stoichiometries of the SRA domain towards differentially modified DNA, 26 µM purified SRA domain in binding buffer (including 100 ng/µl BSA final concentration) was mixed with 20 µM of the corresponding 42bp DNA oligonucleotide in a ratio of 10:1 and incubated on ice for 30 min. The formation of complexes was assessed by size exclusion chromatography on an Aekta Pure system equipped with a Superdex 200 Increase 10/300 GL column. Absorption at 260nm, 280nm and 554nm was monitored to detect DNA, protein and the fluorescence of the oligos’ ATTO550-label.

# References

1. Rothbauer U, Zolghadr K, Muyldermans S, Schepers A, Cardoso MC, Leonhardt H. A versatile nanotrap for biochemical and functional studies with fluorescent fusion proteins. Molecular & cellular proteomics : MCP. 2008;7(2):282-9. doi: 10.1074/mcp.M700342-MCP200. PubMed PMID: 17951627.
